# Analysis of planetary boundaries and economic assessment for waste valorization in the context of a biorefinery: case study of the corn value chain in Sucre, Colombia

**DOI:** 10.1007/s11356-025-36266-x

**Published:** 2025-04-02

**Authors:** Juan Felipe Hernandez-Arango, Mariana Ortiz-Sanchez, Juan Camilo Solarte-Toro, Jairo Salcedo‑Mendoza, Carlos Ariel Cardona Alzate

**Affiliations:** 1 Departamento de Ingeniería Química, Instituto de Biotecnología y Agroindustria, Universidad Nacional de Colombia Sede Manizales, Km 07 vía al Magdalena, Manizales, Colombia; 2 Facultad de Ingeniería, Grupo Procesos Agroindustriales y Desarrollo Sostenible (PADES), Universidad de Sucre, Sincelejo, Colombia

**Keywords:** Life cycle assessment, Corn stover, Simulation, Mulching, Economical comparison, Transgression level

## Abstract

The environmental impact generated by the excessive use of energy and petrochemical products has become a current problem addressed by considering the valorization of waste from a value chain (VC) under the biorefinery concept. At a global level, international organizations have proposed different tools to control the environmental impact of VC. Life cycle analysis (LCA) is the most representative tool. However, the LCA results do not allow defining a VC impact in a territory. The planetary boundaries (PB) approach contextualizes the results of an LCA with the maximum limits allowed for a defined activity. This research aimed to propose and apply a methodology integrating the LCA and PB approach (PBA-LCA). For this, waste valorization under the biorefinery concept was considered. The conceptual process analysis, economic optimization of biorefineries, LCA, and PB approach tools were combined and applied to a representative case study (the corn VC in Sucre, Colombia). First, the corn VC was analyzed to define different valorization alternatives for corn stover (CS). The valorization alternatives were simulated and evaluated using Aspen Plus V9.0., Aspen Economic Analyzer V9.0., and SimaPro V8.3. The LCA impact categories were used to define the PB. The economic optimization of CS biorefineries resulted in the technical and economic limitations of the cellulose valorization fraction due to high capital and operating costs. Moreover, the production of xylitol from CS presented the best economic results with a payback period of 2 years and an NPV of US$26.04 million. The LCA results demonstrated the advantages of using CS in agricultural activities. In the biorefinery, the split of CS scenarios for biorefineries had a higher environmental impact. The inclusion of the valorization stage increases to 5 and 15 times the impact on climate change and freshwater use boundaries, respectively. Finally, the PB results demonstrated the advantage of CS current use. On the other hand, the PB analysis determined the appropriate CS split with a biorefinery/mulching ratio of 70%/30% to be implemented in Sucre, Colombia. In conclusion, the results demonstrated the need to contextualize the results of an LCA with the PB in a given region to analyze the processes’ environmental viability.

**Supplementary Information:**

The online version contains supplementary material available at 10.1007/s11356-025-36266-x.

## Introduction

**Fig. 1:**
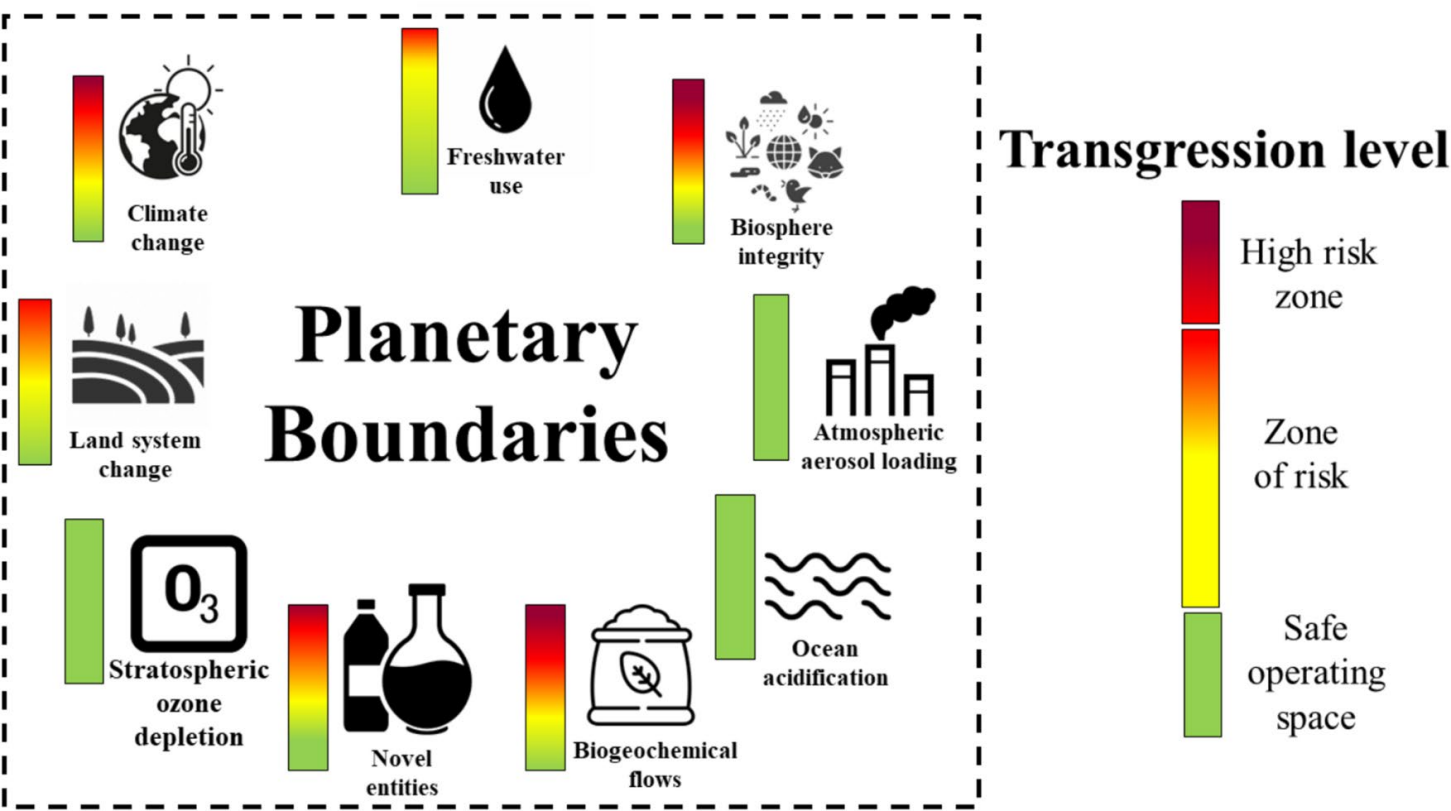
Planetary boundaries (PB) transgression level (TL) for the 2023. Adapted from Richardson et al. (2023)

Environmental impacts caused by the excessive use of fossil fuels and the continued greenhouse gas emissions in the agro-industrial sector have been one of the main problems raised worldwide (Young et al. 2021). Within agronomic activities, the excessive use of fertilizers and agrochemicals (e.g., fungicides, herbicides, pesticides) to increase crop production and meet global demand has been categorized as one of the most relevant problems contributing to environmental damage (Sun et al. 2020). For example, in the case of Colombia, activities related to land use contribute to 59.1% of total national emissions (Karina Enríquez et al. 2018). These issues have increased social inequality, reducing access to basic needs for human well-being (i.e., food, water, education, energy) (Pawlak and Kołodziejczak 2020). Several international organizations have sought different alternatives and approaches to mitigate these challenges based on the development of value chains (VC) (Palmeros Parada et al. 2017). Sustainable development and planetary boundaries (PB) are among the most representative approaches. Sustainable development is “development that meets the needs of the present without compromising the ability of future generations to meet their own needs” (United Nations General Assembly 1987). Moreover, the PB concept was introduced in 2009 to evaluate the environmental impact of human activity globally (Rockström et al. 2009). In 2015, the framework was updated to define the environmental limits where humanity can operate safely and define which boundaries are exceeded (Steffen et al. 2015). These limits were established through 9 representative control variables that are measured on a global scale. Figure 1 presents the PB and transgression level (TL) in the world:

Currently, six PB have been transgressed, putting the earth system at risk. Only the stratospheric ozone depletion, ocean acidification, and atmospheric aerosol loading limits are within the safe operating zone (Richardson et al. 2023). In this sense, it is necessary to propose alternatives from the current model that ensure the subsistence and sustainability of humanity without transgressing the PB (Hauschild et al. 2020). PB establish a framework for measuring the level of environmental impact of human activities in any region. Then, the activities can be analyzed without using comparisons. Consequently, methodologies that combine life cycle analysis (LCA) with PB have been proposed (PB-LCA) (Ryberg et al. 2018a). The main problem with the practical use of PB has been evaluating them in a reduced context. Multiple alternatives have been proposed as presented in Table 1. One route to promote decarbonizing VC and the generation of value-added products considering the PB is biomass valorization (Awasthi et al. 2020). The efficient use of biomass implies introducing the concept of biorefineries, which was born as a sustainable approach to producing multiple products (Culaba et al. 2023). The concept of biorefinery is based on the sustainable processing of biomass for the obtaining of products and energy. Then, biorefinery is defined as a facility that combines technologies to separate and transform biomass (Cherubini 2010). These structures aim to integrate different processes to optimize the use of different biomass fractions (Duan et al. 2022). Several researchers have considered a technical, economic, environmental, and social approach to analyzing the biorefinery scenarios. However, the PB perspective to define the biorefinery impact is not considered.

**Table 1:** Model proposals for implementation of the PB framework

Year	Description	Ref
2009	The PB framework is presented. The 9 limits and some control variables are established, and the level of transgression of the limits is provisionally presented	Rockström et al. (2009)
2012	The PB framework explores social relationships. The problem of applying this framework to regional cases arises. PB analysis is recognized as a guide for governance policies in different regions, and there is a need for greater integration between environmental policies	Nilsson and Persson (2012)
2014	It shows how the PB concept can be applied to regional systems, such as watersheds, national parks, subnational administrative divisions and national states. This type of methodology is used for the case of South Africa	Cole et al. (2014), Dearing et al. (2014)
2015	It explores the relationship between the concept of water footprint, chemical footprint, carbon footprint, and other environmental indicators. It may also create a joint framework for assessing environmental impacts with the PB	Fang et al. (2015)
2015	The PB are reviewed and updated, several limits are updated to values with greater scientific evidence and are established for some new control variables	Steffen et al. (2015)
2018	A case study for linking PB to the ecosystem of the Orinoco River basin in Colombia is presented. It notes that a regional approach would be more useful for local political decision-making and monitoring of environmental variables	Vargas et al. (2018)
2018	A methodology for analyzing the environmental transgression of PB for a specific activity is proposed using the concept of characterization factors and the LCA as a framework for the study of the evaluated activity and the allocation of an SOS	Ryberg et al. (2018b)
2021	A reduction of the PB is made for New Zealand, assessing the country’s environmental impact within this framework	Hoff (2021)
2021	Explore and discuss possible ways to allocate SOS based on principles of equity and responsibility for environmental impact assessment within the framework of the PB in conjunction with LCA	Hjalsted et al. (2021)
2023	The characterization factors applied to the LCA results, as well as the principles of SOS allocation, are used to evaluate the impacts of the European chemical industry	Barnosell and Pozo (2023)

**Fig. 2:**
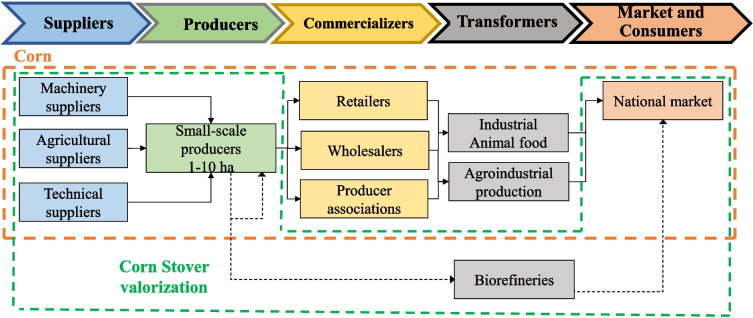
Value chain (VC) of corn in Colombia

Corn VC is of great interest in terms of both scope and volume of production (Qin et al. 2017). This crop has a strong presence worldwide, especially in the USA, China, and Brazil (Aghaei et al. 2022). World corn production is 1.16 billion metric tons (Foreign Agricultural Service 2024). This VC involves the products’ production for human food and livestock feed and higher value-added products like starch and bioethanol. Figure 2 presents a simplified VC for the case of Colombia. Corn stover (CS) is one of the most abundant agricultural wastes worldwide (Khan et al. 2021). CS production is estimated at one billion tons per year (Alavijeh et al. 2023). CS is a lignocellulosic biomass composed mainly of lignin, cellulose, and hemicellulose. According to Zhang et al. (2023), CS composition is 30–60% cellulose, 20–40% hemicellulose, and 15–25% lignin. This composition varies significantly according to the region and cultivation practices (Sluiter et al. 2003). These characteristics are attractive for valorizing in a biorefinery concept (Zhang et al. 2023). Currently, CS is used as poor-quality forage or burned to clear the field, and mulching practices to conserve tillage areas (Alavijeh et al. 2023). Nevertheless, the mulching practice is considered a soil remediation and conservation activity where the main effects are the emission reduction of N_2_O (Hao et al. 2023), erosion reduction (Prasuhn 2020), humidity conservation (Jiménez et al. 2017), and increased mesofauna in the soil (Jiang et al. 2021), among others. Combined with no-tillage practices, these effects have been studied (Wang et al. 2020). The appropriate amount of CS for mulching is estimated at 2.5 tons/ha, equivalent to 30% of the area covered by the hectare (Hao et al. 2023). CS has been proposed as a raw material to obtain energy vectors and value-added products on multiple occasions. Bioethanol production is the most significant proposal and has been implemented on an industrial scale (Ethanol producer magazine 2017). On the other hand, biogas production (Alavijeh et al. 2023), xylitol, levulinic acid, butanol (Zabed et al. 2023), and others have been proposed to valorize the CS. However, these proposals rarely consider the cultural realities of crops, where farmers use CS for other purposes, such as mulching, reducing the availability of CS.

This research proposed a CS biorefinery using an optimization strategy based on a product portfolio according to technological readiness level (TRL), economic viability, and environmental impact in the Colombian context. In addition, the use of CS in mulching practice was considered. For this reason, CS was split considering to approach agricultural remediation and biorefinery to define different scenarios. The raw material was characterized and simulated to define the mass and energy balance using Aspen Plus v9.0. Life cycle assessment (LCA) methodology was applied to calculate environmental indicators. Finally, the environmental impact of the biorefinery was assessed in terms of the PB (PB-LCA) methodology reported by Ryberg et al. (2018a). The approach of linking LCA with PBs has been successfully used to measure environmental impact at different scales (Chen et al. 2021). For instance, this methodology has been used to quantify the environmental impact generated by the petrochemical sector (Galán-Martín et al. 2021). Limitations found in the methodology section include PB yet to be quantified and the allocation methodology. Nevertheless, in the framework of the PB, absolute sustainability indicators can be used to complement the standard metrics of environmental analysis. This methodology has also been used to estimate the environmental impact of consumption in Europe (Sala et al. 2020). In this case, the results indicated the high risk of exceeding the PB with the current consumption model. Thus, advantages were found in linking the LCA with the PB to obtain more accurate conclusions. Despite these results, the integration of the PB-LCA analysis with the techno-economic analysis of biorefineries has not been conducted. The integration of both analyses results in a tool that allows them to contrast the sustainability of the proposed biorefineries. Thus, the aim of this research was to test the use of this methodology applied to a specific biomass valorization context. The statement of novelty of this research can be summarized in the following items.

(i) This research proposes a methodology to evaluate the environmental impact of a biorefinery considering the PB.
(ii) This research proposed a CS biorefinery based on an optimization strategy considering the use of CS as mulching for the Colombian context.
(iii) This research integrated the LCA methodology and the PB perspective.

## Methodology

The impact of different CS use scenarios in PB was analyzed under two approaches: (i) mulching techniques and (ii) biorefinery to obtain value-added products (e.g., xylitol, kraft lignin, among others) and energy vectors (e.g., ethanol, butanol, biogas). The biorefinery scenarios were proposed using the methodology reported by Ortiz-Sanchez and Cardona Alzate (2022). A corn VC perspective in Sucre, Colombia, was considered for the PB analysis. This research was carried out considering the experimental approach and PB analysis. The experimental approach was performed to determine the chemical characterization of CS. These results were used in the simulation biorefineries using Aspen Plus V.9.0. The PB were assessed using the LCA methodology.

### Raw material origin

The CS sample was obtained from Colosó, Sucre, Colombia (9° 29′ 39″ N, 75° 21′ 09″ O). The CS was obtained from yellow corn *Criollo puya* variety (*Zea mays*) under a technified cultivation modality. The raw material was sun-dried until reached a moisture content of 10% on a dry basis. Then, the samples were milled using a knife mill (Gyratory mill SR200 Gusseisen, Redsch GmbH, Germany) until a particle size of 0.45 mm according to the ASTM 40 Mesh (Hames et al. 2008).

### Raw material characterization

The chemical composition was performed to determine the content of extractives in polar and non-polar solvents (i.e., water and ethanol), cellulose, hemicellulose, lignin, and ashes. The standards methodology is presented in Table 2.

**Table 2:** Standards used for CS chemical characterization

Item	Standards	Ref
Extractives	NREL/TP-510–42619	Sluiter et al. (2008b)
Hemicellulose	Sodium chlorite method	Han and Rowell (1996)
Cellulose		
Lignin	TAPPI T222	T.A.P.P.I (2006)
Ash	NREL/TP-510–42622	Sluiter et al. (2008a)

### Planetary boundaries analysis

The PB analysis was carried out based on the methodology proposed by Rybert et al. (2018b). The ISO14040 and 14044 were considered in LCA (British Standard 2006). The environmental indicators obtained in the LCA were contextualized to the PB using characterization factors. For this, a safe operating zone was assigned for the biorefinery activity. The operating zone represents the PB space that biorefinery scenarios have the right to use for its economic and social benefit. The PB analysis aimed to determine whether the CS valorization approaches exceed the PB. A corn VC in Sucre, Colombia, was considered in the LCA.

#### Goal and scope

**Fig. 3:**
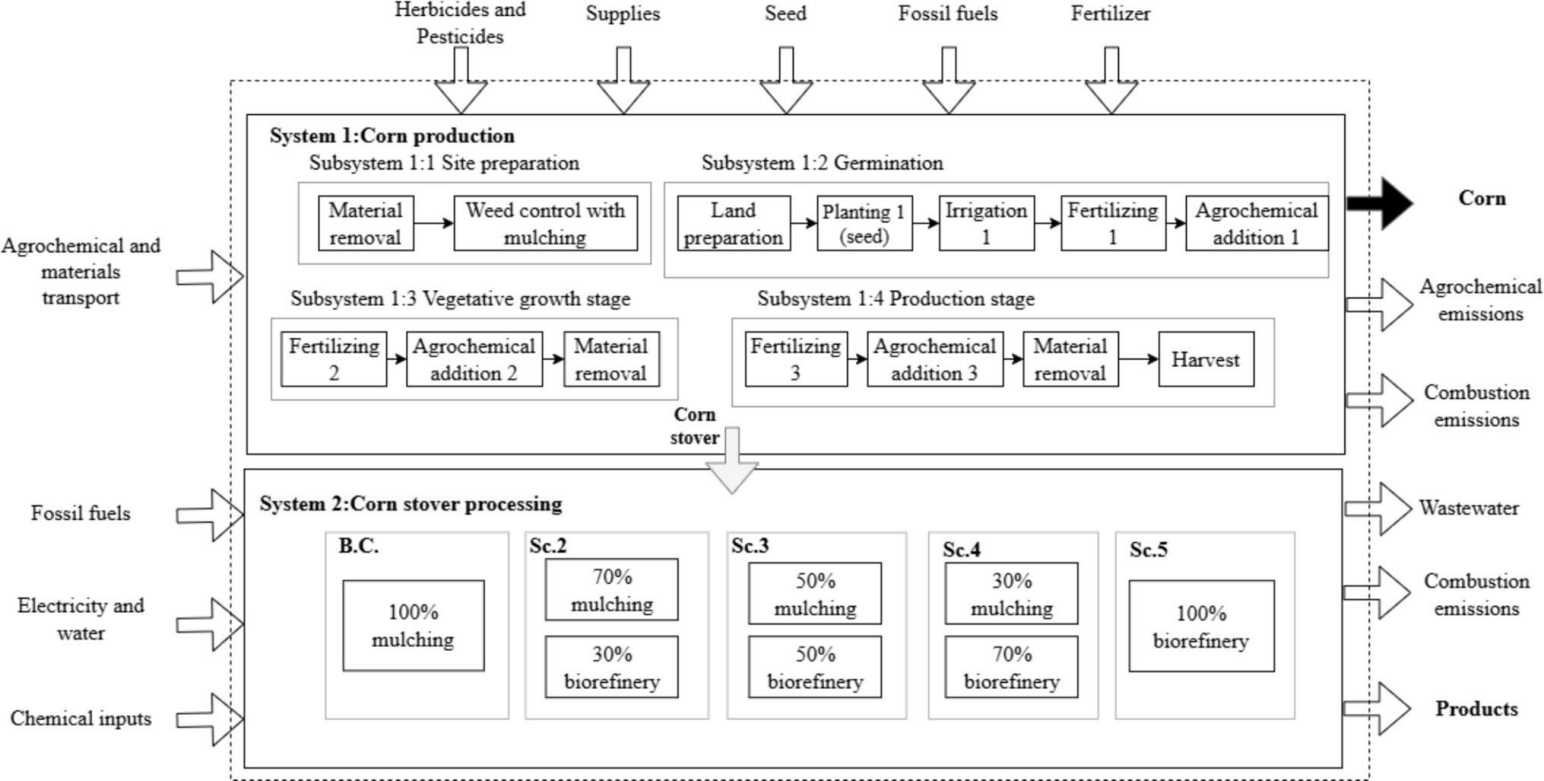
System boundaries of LCA

The goal of the LCA was to evaluate the environmental impact of the corn VC considering the CS valorization as mulching and raw material to obtain value-added products and energy vectors under the biorefinery concept. The system boundaries were set from cradle to gate following an attributional approach. The system boundaries are shown in Fig. 3. The links considered are suppliers, producers, and transformers represented by the biorefinery. The study area was the department of Sucre, specifically the 4 main municipalities with technified crops. The corn crop in the study area has 5910 hectares MinAgricultura 2021), with an average yield of 3.08 tons per hectare (MinAgricultura 2017). CS production can be estimated at a 1:1.2 ratio (corn:CS). Colombia has two harvests per year for 4 months (in September and February) (Peñaranda Gonzalez et al. 2017). For this, a CS flow of 22,080 tons/year was considered, representing 25% of the waste technified origin generated in the region MinAgricultura 2021). CS valorization was carried out using (i) mulching and (ii) biorefinery. The best biorefinery scenario was defined using optimization tools to maximize the economic benefit of upgrading CS. This scenario was used to develop the PB analysis. The functional unit was 1 kg of corn. The scenarios considered are presented in Fig. 3.

The evaluation of the agronomic stage for the LCA is defined by system 1 (see Fig. 3), which includes activities such as site preparation and weed control. Between 30,000 and 80,000 seeds are used per hectare in the germination stage (Fenalce 2011). Then, the irrigation, fertilization (e.g., N, P, and K), and agrochemical (e.g., glyphosate and cypermethrin) applications are performed in this stage. Seed germination usually takes 8 to 10 days. In the vegetative growth stage, the addition of fertilizers and agrochemicals is repeated, with weeding. Finally, the corn harvest takes place between 2 and 3 months.

#### Life cycle inventory

The life cycle inventory (LCI) was carried out considering the links, actors, and activities presented in Fig. 2. The suppliers’ link considered the distances and transport types of agrochemicals and fertilizers. Distances were calculated in Google Maps considering the information provided by VC actors. The inventory of the producers’ link was defined according to the information provided by governmental entities. The LCI of the corn VC for the study region is presented in Table 3. The inventory is formulated per hectare of cultivated corn. The fuel used by the string trimmer is included in the weed control phase. Moreover, the land preparation stage considers the fuel consumption of the tractor. The harvesting stage for the region analyzed is carried out manually. The use of specialized machinery such as combine harvesters is not implemented. The Intergovernmental Panel on Climate Change (IPCC) methodology was used to calculate the emissions (IPCC 2006). The information on the use of agrochemicals was taken from the cultivation manuals available in Colombia (Fenalce 2011).

**Table 3:** Life cycle inventory of producer link of corn VC

Activity	Inlet			Output		
	Item	Value	Unit	Item	Value	Unit
Suppliers						
Agrochemical and fertilizer transportation	Transportation distance Corozal-Coloso^*^	48.5	km			
Small producers						
Irrigation	Water	500	L	-		
Agrochemical addition	PANZER 480 SL^**^	1	L	-		
	Invetrin^***^	1	L			
Weed control	String trimmer oil use	2	L	-		
	String trimmer gasoline use	14	L	CO_2_	28.21	kg
				CH_4_	0.97	g
				N_2_O	0.96	g
Land preparation	ACPM for plough	20	L	CO_2_	54.38	kg
				CH_4_	0.20	g
				N_2_O	0.20	g
	ACPM for hilling	20	L	CO_2_	54.38	kg
				CH_4_	0.20	g
				N_2_O	0.20	g
Fertilizing	Nitrogen	120	kg	NO_3_	36,000	g
				NH_3_	2914.28	g
				Direct N_2_O	1885.71	g
				Indirect N_2_O	197.14	g
	Phosphorus	80	kg	P^*^ emissions to soil	4259.61	g
	Potassium	160	kg	-		
	Sulfur	0.1	kg			
	Sodium	0.1	kg			
Manual harvest	-			Corn	3.08	Ton
				Corn stover (CS)	3.67	Ton

^*^Calculated with Google Maps

^**^Glyphosate 480 g/L

^***^Cypermethrin 200 g/L

The inputs and outputs of agricultural remediation by mulching were obtained in the open literature. This activity shows evidence for soil conservation for reducing erosion and emissions (applied in the long term) (Hao et al. 2023). Although the effects of mulching are more complex, in this research, the analysis was limited to the emissions. For this, the beneficial effects obtained from mulching application per hectare were estimated concerning emissions. The reductions caused by mulching are presented in Table 4.

**Table 4:** Life cycle inventory of mulching activities

Category	Amount of CS kg/ha	Effect	Ref
CO_2_ emissions	8000	Reduction 5%	Hao et al. (2023, Wang et al. (2020)
N_2_O emissions	2500	Reduction 55%	
	5000	Reduction 38%	van Kessel et al. (2013), Hao et al. (2023), Fan et al. (2018)
	7500	No change	
	10,000	Increase 25%	

**Fig. 4:**
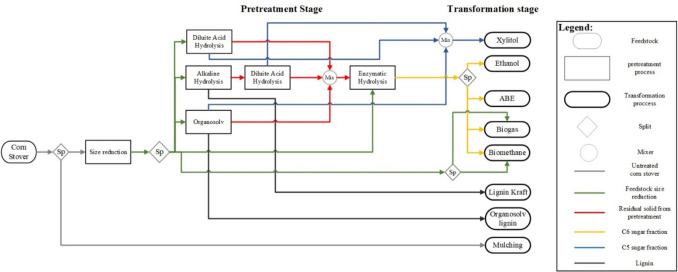
CS biorefinery superstructure

The LCI for the biorefinery transformation link was obtained from process simulation. The biorefinery scenarios were proposed using the methodology reported by Ortiz-Sanchez and Cardona Alzate (2022). The methodology is based on filters to delimit the most viable products. The methodology contemplates five steps: (i) define the goal; (ii) select bioprocesses from the TRL and the degree of industrialization in the study zone; (iii) filter bioprocesses according to technical, economic, and environmental indicators; (iv) define the biorefinery scenarios according to design strategies; and (v) evaluate the sustainability of biorefinery scenarios. The goal of this research was to evaluate CS biorefineries considering the best performance in economic and environmental terms. In this sense, the evaluated zone has a low technological level. Therefore, only products with a high TRL were considered. Economic and environmental indicators weighed 40% and 60%, respectively. This selection was made based on the importance of PB compliance. The selected bioprocesses were biogas and digestate, biomethane and digestate, ethanol, acetone-butanol-ethanol (ABE), xylitol, and kraft lignin. A superstructure was formulated following the conceptual design methodology, which included the use of hierarchy and sequencing (Aristizábal Marulanda et al. 2019). The superstructure is presented in Fig. 4.

Superstructure presents pretreatment steps that consider different raw material fractions and bioprocess routes to obtain added-value products and energy vectors. The CS superstructure was optimized in economic terms. The objective function is presented in Eq. 1. The optimal configuration of the biorefinery was founded on formulating a mixed-integer programming (MIP) problem solved using GAMS (General Algebraic Modeling System) as a computational tool, using CPLEX as a solver (Aristizábal Marulanda et al. 2019). The pretreatment stage and bioprocess in Fig. 4 were simulated using Aspen Plus V9.0. For the complete superstructure, a processing scale of 22,080 tons/year of CS was used. This scale corresponds to the total biomass available in the region to be used in the biorefinery by not considering the current use as mulching. The equipment cost was obtained in Aspen Economic Analyzer V9.0. The capital expenditure (CapEx) and operational expenditure (OpEx) were calculated using the methodology reported by Peters et al. (2002). Equipment costs were updated using the Chemical Engineering Plant Cost Index (CEPCI). Other costs including equipment installation, instrumentation and control, piping (installed), electrical (installed), buildings including services, yard improvements, service facilities (installed), engineering and supervision, construction expenses, legal expenses, contractor’s fee, and contingency were estimated following a detailed conceptual design methodology (Rueda-Duran et al. 2022). The utility, labor, and chemical reagents costs are presented in the Supplementary Material Table S1.

1 $$NPV=\sum_{i=1}^{n}\frac{{CsF}_{i}}{{(1+ir)}^{i}} -{C}^{inv}$$

where ${CF}_{i}$ is the cash flow for the year *i*, $ir$ is the interest rate of the project, *n* is the life of the plant, ${C}^{inv}$ is the fixed capital investment, and the NPV is the net present value. The mass and energy balances of this biorefinery scheme with the best NPV were used as the inventory data for the biorefinery transformation link. The information is presented in Supplementary Material Table S2.

#### Life cycle evaluation

Life cycle evaluation (LCE) was performed using SimaPro 8.3 software and Ecoinvent 3 database (detailed in the Supplementary Material (Table S3)). The ReCiPe midpoint method (Hierarchist version) to determinate climate change, ozone depletion, photochemical oxidant formation, natural land transformation, water depletion, freshwater eutrophication, and marine eutrophication was considered.

#### Interpretation

The LCE results were used to assess the impact of the CS biorefinery scenarios on PB according to the methodology reported by Ryberg et al. (2018a). The LCA results were used by applying characterization factors (CF) to transform the impact categories into PB control variables expressed in time (PB-LCA) (Ryberg et al. 2018b). The step from LCA indicators to impact on control variables (ICV) for each PB was calculated using Eq. 2 (Ryberg et al. 2018a). CF are specific to each planetary boundary and impact category of the LCA. Nevertheless, the same LCA indicator can have an impact on more than one planetary boundary. For instance, the climate change indicator (CO_2_ equivalent emissions) has an impact on energy imbalance, CO_2_ concentration, and ocean acidification boundaries. The specific choice of the CF was made according to the PB evaluated and the impact categories calculated using the ReCiPe midpoint method.

2 $$IC{V}_{n}=C{F}_{n}.LC{I}_{n}$$

LCI is the LCA result for the impact category *n*. The CF for the six PB is provided by Ryberg et al. (2018b). Safe operating spaces (SOS) are the impact space intended for human subsistence. This space is defined globally as the PB minus the space nature occupies for human subsistence. For example, in the case of climate change, the limit is 350 ppm CO_2_. Nature subsistence occupies 278 ppm, leaving 72 ppm as SOS for humanity. The SOS for humanity is shown in Table 5.

**Table 5:** Secure operating space for humanity. Adapted from Ryberg et al. (2018a) and Steffen et al. (2015)

Impact category	Unit	Safe operating space for humanity
Climate change, energy imbalance	Wm^−2^	1
Climate change, CO_2_ concentration	Ppm CO_2_	72
Stratospheric ozone depletion	DU	15
Ocean acidification	Mol	0.69
Biogeochemical flows, P global	Tg P yr^−1^	6.2
Biogeochemical flows, N global	Tg N yr^−1^	62
Land system change, global	%	25
Land system change, boreal	%	15
Land system change, tropic	%	15
Land system change, temperate	%	50
Freshwater use, global	Km^3^ yr^−1^	4000
Atmospheric aerosol loading	AOD	0.11

*Wm*^*−2*^, watts per cubic meter; *Ppm*, parts per million of CO_2_; *DU*, Dobson unit; *mol*, mol to aragonite; *Tg yr*^*−1*^, teragram per year; *%*, percentage of change; *Km*^*3*^* yr*^*−1*^, cubic kilometer per year; *AOD*, aerosol optical depth

This global operating space can be expressed in regional terms to facilitate the evaluation of the environmental impacts. Human activities are entitled to an equitable and fair share of the SOS. Allocation should be based on principles of equity, responsibility, and cost-effectiveness (Tulus et al. 2021). For example, every person has the right to use the planet resources for subsistence also following the principle that greater economic value promotes greater well-being. Activities that benefit larger populations or generate greater economic profitability should have a larger area of the SOS. In this research, sensitive analysis was performed considering the two most common types of allocation. Table 6 presents the SOS allocation used in CS biorefineries.

**Table 6:** Criteria for SOS allocation (Ryberg et al. 2018a)

Allocation criteria	Description
$SO{S}_{n}=SO{S}_{T}*\frac{{P}_{a}}{{P}_{world}}$	SOS_n_: Safe operating space of the activity *n* SOS_T_: Safe operating space total *P*_world_: population of the world *P*_*a*_: population of the region *a*
$SO{S}_{n}=SO{S}_{T}*\frac{GD{P}_{n}}{GD{P}_{world}}$	GDP_world_: gross domestic product of the world GDP_n_: gross domestic product of the activity *n*
$SO{S}_{n}=SO{S}_{T}*\frac{{P}_{country}}{{P}_{world}}*\frac{GD{P}_{n}}{GD{P}_{country}}$	*P*_country_: population of the country GDP_country_: gross domestic product of the country

*n* represents the value chain of corn for a: the region of Sucre in Colombia

The per capita allocation was considered the first criteria where the SOS is distributed equally among all humanity. The SOS was defined based on the people who can benefit from the specific activity. The second criterion included the economic factor to give more weight to those activities that generate a major economic benefit. The third allocation considered both the population and the economic importance of the activity evaluated. These types of allocation criteria are the most common but have some limitations. A criterion based on population density ignores that industries and points of economic activity may be concentrated in a specific region. Then, regions with high economic density but low population could have a smaller SOS than would be fair. Another limitation of these allocation criteria is the assumption that a VC generating large economic benefits also generates better living conditions. There are cases where the search for cost reduction and profit maximization generates negative impacts on the quality of life (Surmeier et al. 2024). Thus, caution should be taken when using economic indicators as a reflection of quality of life. The use of a combined criterion reduces the uncertainty of each allocation method but does not solve the problems exposed.

The SOS_n_ allows interpretation of the results of Eq. 2 derived from the LCA. The ICV for each control variable must be within the assigned SOS. The SOS_n_ results were used to define the impact of ICV. In this sense, the ICV of the CS biorefinery scenarios must be within the values defined for TL (see Eq. 3) (Tulus et al. 2021). Values of TL greater than one were considered to exceed its SOS. Values less than 1 represented an operation within the safe operating zone. Supplementary material S1 presents a sample for the calculation of impacts following the PB-LCA methodology.

3 $$T{L}_{n}=\frac{IC{V}_{n}}{SO{S}_{n}}$$

## Results and discussion

### CS characterization

The CS chemical composition on a dry basis is presented in Table 7 together with other results reported in published research articles. The high content of cellulose, hemicellulose, and lignin demonstrates the potential of CS to be used as a raw material in biorefineries. The CS valorization by ethanol fermentation has been mainly proposed (Li et al. 2016). However, the production of butanol, hydrogen, biogas, lactic acid, succinic acid, xylitol, citric acid, itaconic acid, and 1–3 propanediol have been considered (Zabed et al. 2023). In the biorefinery concept, CS has been proposed to produce chitosan, methane, biodiesel, bioethanol, glycerin, and animal feed (Alavijeh et al. 2023). Other configurations have also been proposed, including ethanol, acetic acid, phenol, furfural, cresols, catechol, formic acid, and acetaldehyde (Bbosa et al. 2018). However, the products obtained from the valorization of CS are not contextualized with the region where the waste was generated.

**Table 7:** CS characterization

Item	Fraction			
Reference	This work	Zhao et al. (2014)	Berchem et al. (2017)	Noppawan et al. (2021)
Water extractives	14% ± 0.98	–	11.0	–
Ethanol extractives	4.8% ± 0.63	–	3.3	–
Cellulose	28.9% ± 1.11	46.2	37.5	38.1
Hemicellulose	21.3% ± 0.82	28.0	18.7	25.3
Lignin	25.6% ± 2.32	10.6	13.4	20.2
Ash	4.9% ± 0.21	6.5	5.7	4.84
Others		8.7	4.7	–

The chemical composition reported in the open literature of CS differs significantly considering aspects such as the variety and the growing region. For example, Zhao et al. (2018) report a higher cellulose and hemicellulose content and a lower lignin content compared to the results obtained in this research. Öhgren et al. (2007) also reported higher cellulose and lower hemicellulose contents for European and USA corn. However, García Stepien et al. (2017) reported similar results for Latin American corn. The CS composition of European origin (Öhgren et al. 2007) and American (Kim et al. 2003) have a higher content of cellulose and hemicellulose. The lignin content can be explained by the corn variety, corresponding to the “Puya” typical of Colombia (René and Cardona 2014). This variety is also present in the border areas with Venezuela and has taxonomic similarities with other varieties present in Ecuador (Serratos Hernández 2009). On the other hand, the composition of CS of European origin (Öhgren et al. 2007) and American presents predominant compositions in terms of cellulose and hemicellulose contents. This characteristic makes it more difficult to separate the lignocellulosic fractions, implying the need to use aggressive pretreatment conditions or longer residence times (Zhuo et al. 2018).

### Optimization results

**Fig. 5:**
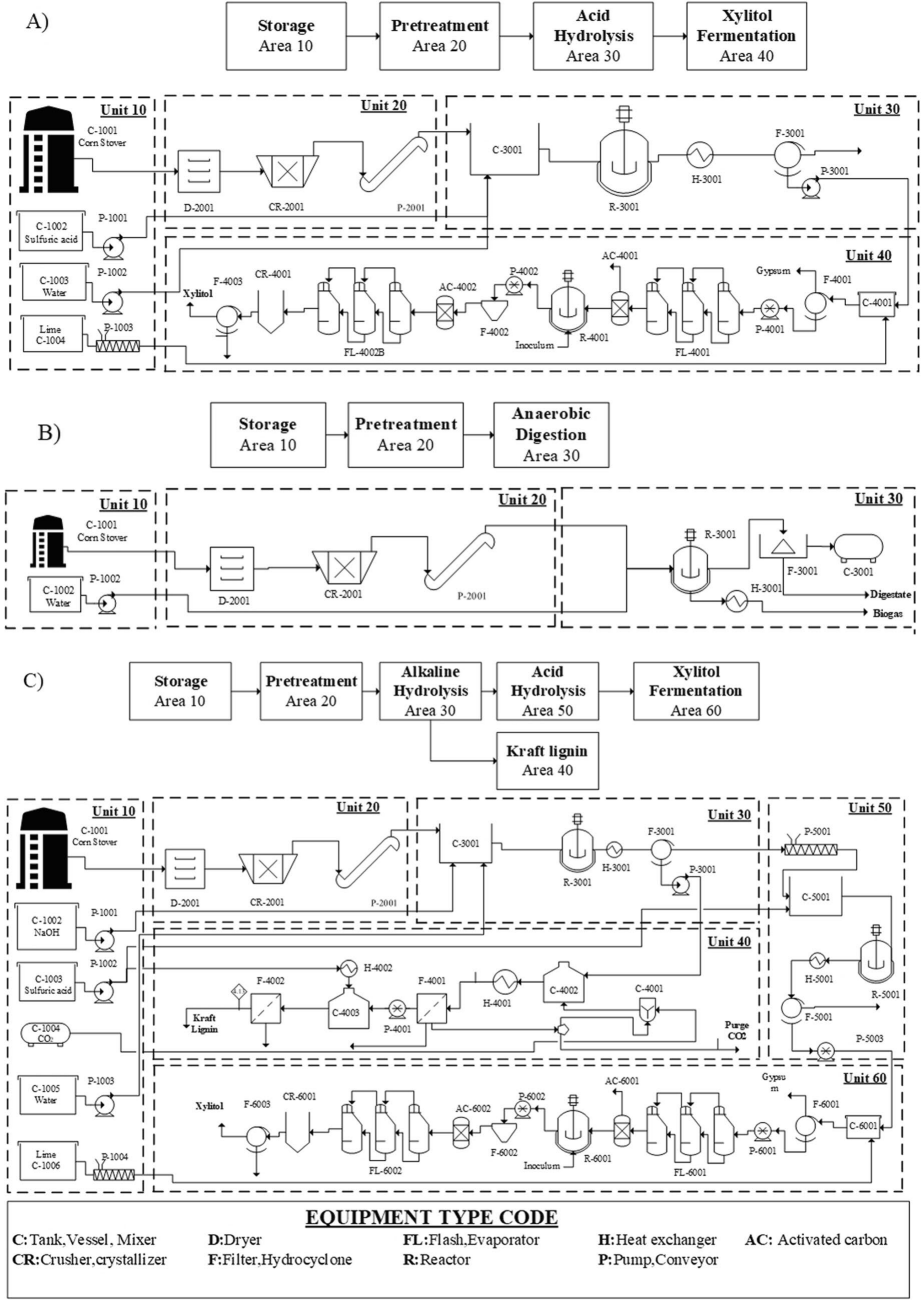
Process diagram for the biorefinery. **A** Acid hydrolysis and xylitol. **B** Anaerobic digestion. **C** Alkaline hydrolysis, lignin kraft, acid hydrolysis, and xylitol

The results of the economic optimization of the CS superstructure more economically feasible were as follows: (i) alkaline hydrolysis, lignin kraft, acid hydrolysis, and xylitol with NPV of 6.8 mUSD and payback period of 7 years; (ii) alkaline hydrolysis, acid hydrolysis and xylitol with NPV of 4 mUSD and payback period of 8 years; (iii) alkaline hydrolysis, acid hydrolysis and anaerobic digestion with NPV of 0.2 mUSD and payback period of 17 years; (iv) anaerobic digestion with NPV of 12.7 mUSD and payback period of 1 year; (v) acid hydrolysis and xylitol with NPV of 26 mUSD and payback period of 2 years. The process flow diagram of the 3 biorefineries with the highest economic pre-feasibility is shown in Fig. 5. Biorefinery A showed a yield of 0.17 kg xylitol/ kg CS which is in agreement with the literature (Shaji et al. 2022). The main differences can be attributed to the difference in the composition of the Colombian CS. This result is better than the one presented by Biorefinery C with a moderate yield of 0.09 kg xylitol/ kg CS and 0.14 kg kraft lignin/kg CS. The inclusion of an additional pretreatment causes the loss of the hemicellulose fraction which decreases the xylitol yield. Nevertheless, this allows the valorization of the lignin fraction. On the other hand, Biorefinery B has a yield of 0.72 m3/kg CS. This result is within the ranges obtained for biomass valorization by anaerobic digestion (Kasinath et al. 2021).

The CS biorefineries including cellulose valorization from enzymatic hydrolysis were discarded. The high enzyme costs and the high residence times required in cellulose hydrolysis caused economic unfeasibility in the CS biorefineries. In addition, the scale of CS processing is low compared to that recommended to produce energy vectors. (Leboreiro and Hilaly 2011). For this reason, the CS biorefineries presented in Fig. 5 did not include cellulose valorization. However, chemical pretreatments with acids and bases showed higher pre-feasibility as they required cheaper inputs and shorter residence times. (i.e., enzymatic hydrolysis of cellulose required 72 h, alkaline hydrolysis required 2 h, and acid hydrolysis required 3 h). The main solid wastes from biorefinery A and C were the cellulose fraction. This fraction can be valorized using low-complexity processes such as composting. CS composting has been reported yields of 16% of organic fertilizer (Romero-Figueroa et al. 2015). Nevertheless, further research should be conducted to determine the feasibility of this route.

**Fig. 6:**
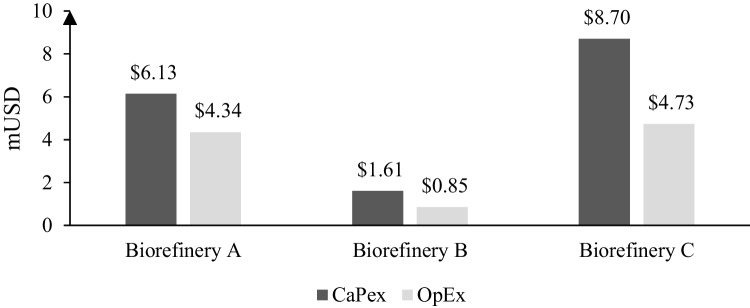
Capex and OpEx of integrated schemes

The scenarios presented in Fig. 5 were energetically integrated among the different units to reduce operating costs and achieve higher viability. The economic results in terms of CapEx and OpEx are presented in Fig. 6. Biorefinery C had the highest investment cost and involved the largest operating units. Biorefinery B did not require the use of high-level technologies. For this, CapEx costs were lower. Pretreatment of biomass or little recurring costs, demonstrating that anaerobic digestion is easily implemented and inexpensive. On the other hand, the greatest impact on OpEx for scenarios A and C was due to the use of steam, which represents 52.5% and 32.5% of the total OpEx. This is mainly due to the sugar concentration stages and the xylitol purification before crystallization. In scenario B, the main contributor was labor with 36.5% followed by energy requirements with 29.4%.

**Fig. 7:**
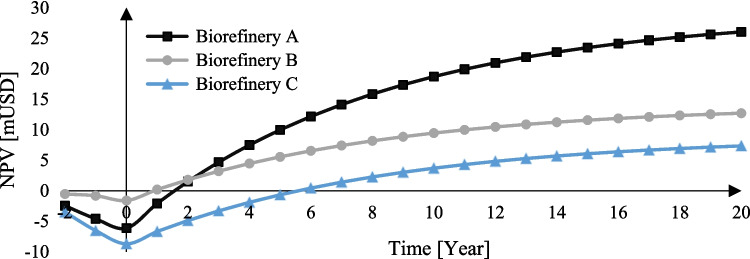
Net present value (NPV) of biorefinery schemes

Figure 7 shows the economic feasibility results for the CS biorefineries. Biorefinery A with acid hydrolysis and xylitol had the highest NPV, with a payback period of 2 years. The high economic profitability of this biorefinery was for the high selling price of xylitol; in addition, the use of few pretreatment steps prevented the loss of the hemicellulose fraction, which allowed a higher utilization of these sugars. Moreover, this scheme was susceptible to the selling price of xylitol to ensure the economic feasibility. The next best result was in biorefinery B where the anaerobic digestion required a lower CapEx due to the few processing units and a higher TRL compared to the other biorefineries. This economic prefeasibility was reflected in a low payback period (1 year). The NPV of biorefinery B was 48.24% compared with biorefinery A. Finally, the production of xylitol and kraft lignin (i.e., biorefinery C) had the lowest results, obtaining a final NPV representing only 28.24% compared to scenario A. On the other hand, scenario C presented the highest return period with a value of 6 years. This was because a greater number of process steps were used, increasing the CapEx as shown in Fig. 6. In addition, the sequence of two chemical pretreatments caused a greater loss of the hemicellulose fraction usable in later stages of the process. However, it is still profitable and can be used as a base case in the search for the valuation of this fraction, such as the production of vanillin by the oxidation route. The results determined the use of biorefinery scheme A for PB analysis.

### Life cycle assessment results

Table 8 presents the LCA results. The base scenario (corn harvesting and the use of the CS as mulching) had a climate change category of 0.49 kg CO_2_ eq/kg of corn harvested, where the use of fertilizers contributed 94.71%. Li et al.(2021) reported the range of 0.281 to 0.814 kg CO_2_ eq/kg of corn, with the main contribution also due to nitrogen fertilizers. This is an expected result as the corn crop requires high nitrogen fertilization, but some of the nitrogen supplied is lost by different mechanisms (Jankowski et al. 2018). In countries like China, the fertilizers used generate 39% of the total emissions in corn production, considering the application dose of 200 kg N/ha (Zhang et al. 2017). The cultivation phases require strategies to minimize nutrient loss and increase fertilization efficiency, which decreases the environmental impact, Moreover, the leaching of N and P fertilizers can endanger the water sources causing eutrophication, which is a typical problem in cultivated areas (Balasuriya et al. 2022). For this research, the impact categories of Marine eutrophication and Freshwater eutrophication were found in the same range as those reported for corn crops in the USA (Lee et al. 2020).

**Table 8:** Environmental impact of the scenarios analyzed

Impact categories	Units	B.C	Sc2	Sc3	Sc4	Sc5
Climate change	kg CO_2_ eq	0.49	2.50	2.66	2.71	2.87
Ozone depletion	kg CFC-11 eq	4.77E − 08	2.54E − 07	2.54E − 07	2.54E − 07	2.54E − 07
Freshwater eutrophication	kg P eq	2.09E − 03	2.15E − 03	2.15E − 03	2.15E − 03	2.15E − 03
Marine eutrophication	kg N eq	2.40E − 03	3.18E − 03	3.18E − 03	3.18E − 03	3.71E − 03
Water depletion	m^3^	0.02	0.30	0.30	0.30	0.30
Photochemical oxidant formation	kg NMVOC	2.25E − 03	6.97E − 03	6.97E − 03	6.97E − 03	7.10E − 03
Natural land transformation	m^2^	3.79E − 04	8.63E − 04	8.63E − 04	8.63E − 04	8.63E − 04
Fossil depletion	kg oil eq	0.12	0.77	0.77	0.77	0.77

Scenario 5 obtained an increase in the climate change category with a value of 2.86 kg CO_2_ eq per kg of corn, where energy requirements in the transformation stage with steam generation contribute 75.36% and the agronomic phase contributes 22.71%, This demonstrates how using pretreatments with high dilution rates to release C5 sugars significantly contributes to the increase in emissions associated with the process. A 65% increase in the marine eutrophication category was also observed due to increased nitrogen losses in the cultivation stage when CS was not used as mulching. The other scenarios presented intermediate results between B.C and Sc5.

### PB analysis

**Fig. 8:**
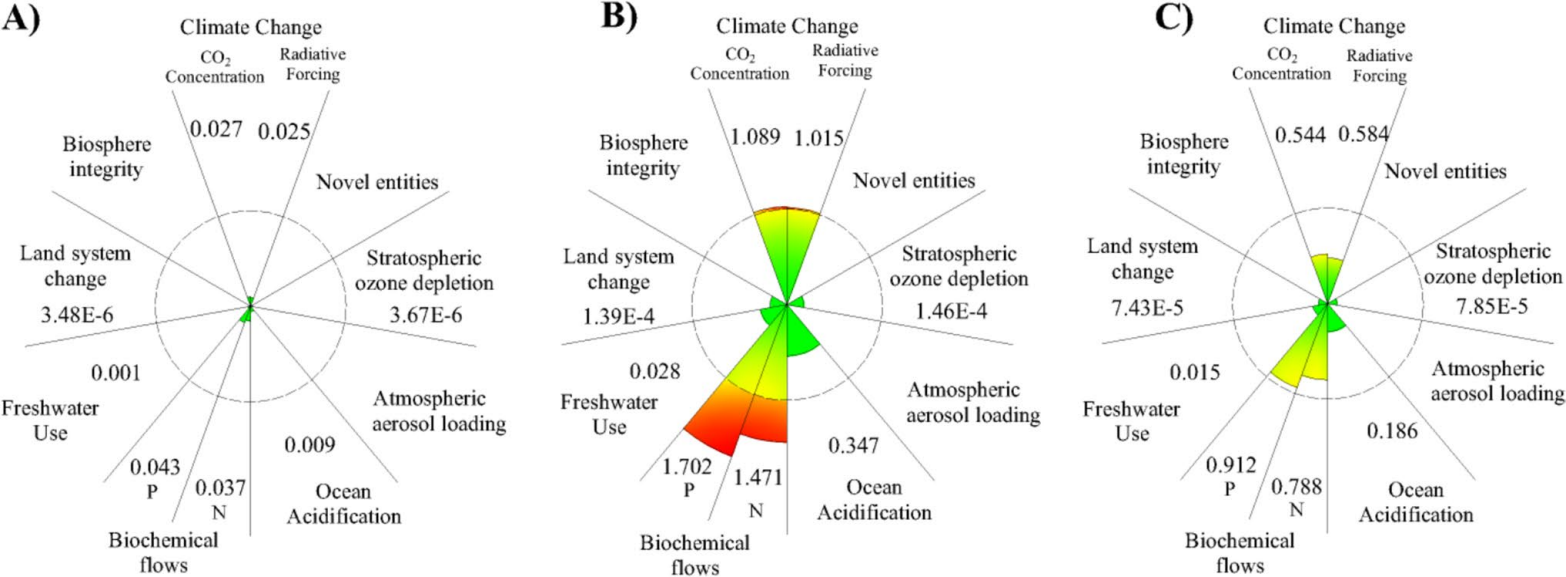
The results of transgression level in PB of base case. **A** Allocation per capita. **B** Allocation GDP activity. **C** Combined allocation

The results of the TL in PB for corn crops are presented in Fig. 8.

**Fig. 9:**
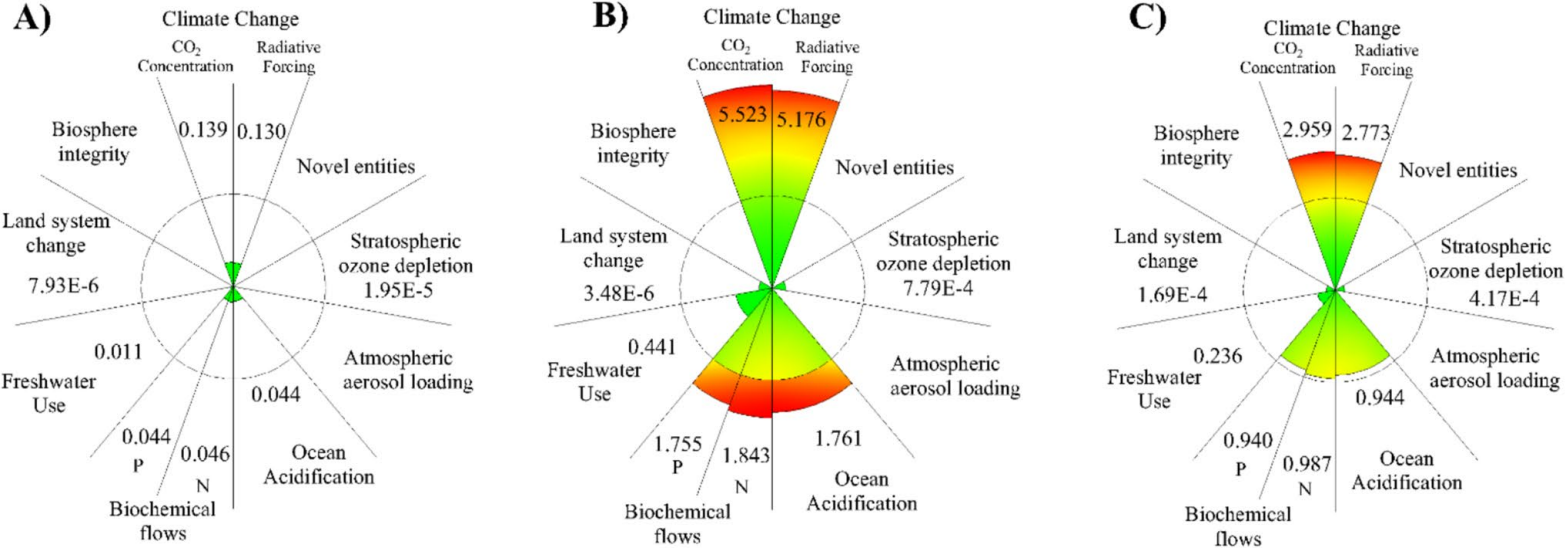
The results of transgression level in PB of CS valorization. **A** Allocation per capita. **B** Allocation GDP activity. **C** Combined allocation

The results of the TL in PB for CS valorization in a biorefinery context are presented in Fig. 9. In the allocation by population, none of the PBs was in a risk zone. In contrast, in the allocation by economic indicators, 3 of 6 PB analyzed were considered surpassed in the valorization scenario, and 2 in the base case of the VC analyzed. An intermediate result was found in the allocation combining population with economic criteria, whereas one limit was exceeded in the valorization scenario. In the base case, none was exceeded. These differences in results show that the validity of an analysis using the PB-LCA model should be concentrated to reduce the subjectivity of the allocation stage for SOS calculation. In the agricultural phase, it was found that the most affected PB are mainly biochemical flows, ocean acidification, and climate change. This is in line with the main problems identified in the current agricultural cropping model where the use of fertilizers together with the associated emissions are the main problem that makes it currently unsustainable (Ahmed et al. 2017). On the other hand, the inclusion of the CS valorization stage causes an important increase in the impact generated by the activity. Biorefinery causes a more than 5 times increase in TL for the climate change and ocean acidification boundaries and a 15 times more increase on the freshwater use boundary. Moreover, a 25% increase in the biochemical flow of N was obtained. The biorefinery requires a high energy content for the operation, mainly the evaporation stages. Then, the utilities needed for the operation caused 75% of the impact on the climate change boundary. Furthermore, high dilutions required for the acid pretreatment stage cause considerable water needs. This contributes 90% of the TL to the freshwater use boundary. Water recirculation can be considered; however, inhibitors and other impurities are formed and can accumulate during acid hydrolysis. Another alternative would be to use other types of pretreatments where lower dilution rates are required. Finally, a decrease on the quantity of water used in the process would also reduce the utilities required, thus improving the environmental performance of the biorefinery.

The TL results obtained for the corn VC in the base case and CS biorefinery show significant differences between per capita population and GDP allocations. However, the TL results that combine per capita population and GDP allocations present partial results. The per capita allocation was easy to use because it only required census data but presented subjectivity in delimiting the population affected by the activity under evaluation. This is because of the reliability of the data acquired in the inventory phase of an LCA. Population data are uncertain in countries like Colombia, which has hard access areas (e.g., the Sucre region). Therefore, this type of allocation can increase the subjectivity of the results. Another difficulty that per capita population allocation presents is that there is not always equity among the regions. From this, the principle of equal sharing of environmental space for human development would not be satisfied. Allocation using GDP ensures that the most economically important activities receive a larger footprint.

Similarly, the countries contributing the most to the world GDP receive more space from the SOS. On the other hand, the environmental burden of the activities carried out in a CV must be distributed considering the profits of each actor and link. The allocation that combines both criteria partially solves the problems mentioned above. This allocation is not a general per capita allocation because, within country-level systems, it is easy to obtain statistics on the percentage impact of existing VC on the national economy. In addition, using a per capita allocation followed by one according to economic indicators prevents the reduction of the impact space for developing countries. From this, it is possible to reduce some subjectivity when using these combined allocations, but it will continue to depend on the quality of the information available.

**Fig. 10:**
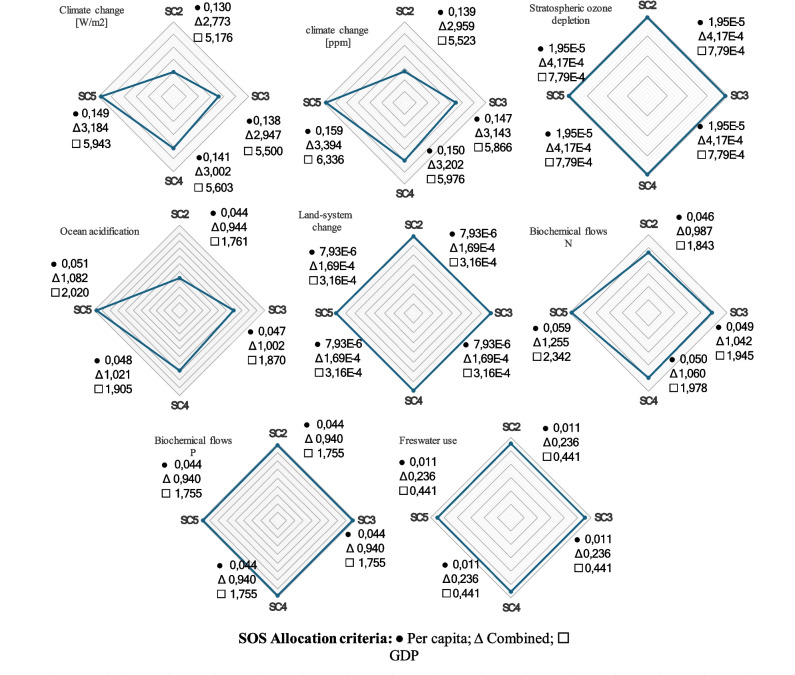
Transgression level (TL) variation for each scenario. The vertices represent the TL value. The vertices closer to the center have less impact on the PB compared to the other scenarios

Figure 10 presents a comparison between the allocation methodologies used. The three types of allocation give substantially different results in numerical terms, but the TL results present a similar tendency. However, the variation in values caused by the different methodologies has a very significant impact on the final conclusions. For instance, in the case of the climate change boundary, a per capita allocation concludes that the activity is within the SOS. Nevertheless, in the case of the other two methodologies considered, 3 to 5 times more than the assigned SOS exceed the boundary. These significant differences indicate the importance of contrasting the results with other methodologies. The use of unique data can produce conclusions that do not really reflect the environmental impact of the activity analyzed. Allocation based on a per capita criterion results in a larger SOS than the other cases. Finally, the use of GDP criteria restricts the SOS. In order for the PB-LCA results to be used properly, the possible differences caused by the selected methodology must be considered. In all cases, the PB of climate change and biochemical flows are the most impacted in the current state of VC, while in the CS valorization scenario, climate change and ocean acidification boundaries are the most impacted compared to the base case. This is explained by the high requirement of utilities in biorefineries, which is reflected in the production of greenhouse gases in the boiler.

Using agricultural remediation practices such as mulching also proved to have a positive impact by reducing the impact generated mainly in the PB of climate change, ocean acidification, and biochemical flows. However, the effectiveness of this impact is determined by the CS application rate used in the field, as shown in Fig. 10. Sc.2 showed the best results by achieving a decrease in TL in several of the PB analyzed; for example, a comparison between Sc.2 and Sc.5 in the PB of climate change, ocean acidification, and biochemical flows of N shows a decrease of 12.90%, 12.82%, and 21.32%, respectively. At the same time, the other scenarios considered showed intermediate results. On the other hand, the other PB analyzed showed no significant differences between the scenarios studied. Mulching practice has a minor impact on the stratospheric ozone depletion and land system change boundaries. Moreover, the freshwater use depends on the climatic conditions of the crop and the local practices since the influence of mulching on these aspects is weak. The main variations will come from the context of the crop analyzed, the level of technology, and other agricultural practices used. This allows to conclude that mulching does not significantly impact these PB. The LCI depends entirely on the country, agricultural practices, and other socio-economic considerations (Bessou et al. 2013). Thus, the impact quantification may vary depending on the context analyzed.

Mulching practices have been shown to have positive impacts but also generate limitations when using CS as a raw material for biotechnological processes. However, scenario 2 has the best environmental results because it requires a higher percentage of CS for mulching, leaving less available for valorization. This may cause logistical problems by requiring CS to be gathered from more regions to meet the demands of the proposed biorefineries. However, these conclusions depend strongly on the region analyzed because Corn and CS yields vary significantly depending on the country and region analyzed. For example, countries such as the USA, with an average yield of 11 tons/ha of corn, will easily meet the CS demands necessary to be used in the mulching practice. Excess CS can be used in biorefineries for valorization. Moreover, environmental benefits of mulching would be obtained at the agricultural stage, and the economic benefits of CS valorization would be also achieved. This solution is the most sustainable by achieving success with both objectives. In contrast, countries or regions with low yields, such as the Sucre region, have an average yield of only 3 tons/ha of corn, which will require the great majority of the CS produced to meet mulching needs. In this case, an environmental benefit would be impossible to achieve through the implementation of the proposed biorefineries. Nevertheless, the implementation of a valorization scheme for CS as mulching and raw material requires logistical coordination with farmers and industries. The biorefinery proposed for the valorization of CS produced a PB transgression five times higher than the traditional use in terms of GDP allocation and three times higher in terms of combined allocation. For better results, biorefineries should implement a portfolio of products economically viable with the scale of CS available. In similar conditions to the Sucre region, where biomass is low available, low-scale processes are a priority to avoid interfering with existing environmental practices. Thus, other biomass and residues can be considered if availability is not sufficient. However, the social and economic benefits generated by the proposed biorefinery must be considered to evaluate the sustainability of the bioprocess.

## Future perspective

The valorization of CS into different products such as xylitol and biogas are economically viable alternatives. These processes can be improved, mainly by implementing pretreatments to achieve high accessibility to the sugar fractions without requiring high dilution rates. The decrease in the quantity of water would improve the technical and economical results of the process by requiring less energy in the evaporation stages. Moreover, the decrease in steam used would have positive consequences in the environmental performance. Further analysis considering technologies that achieve these objectives would be useful to establish the best process configuration. However, the cultural practices of the CS may limit the availability of this waste. For these cases, low-scale valorization alternatives should be studied to determine the viability. Studies detailing in depth the technical, economic, and environmental complications of using CS in biorefineries and as mulching are needed. The PB-LCA methodology permitted to analyze the impact generated by a biorefinery scheme. Additionally, these results were compared with the current use of the lignocellulosic residue. However, strong variations were observed in the results according to the SOS allocation methodology used. Future work should focus on proposing alternatives to reduce uncertainty at this stage. Then, more accurate indicators to precisely describe the social and economic context of the analyzed region would be the best proposals.

## Conclusion

For the first time, a methodology combining conceptual design, LCA, and PB approaches was proposed for biorefineries. This methodology elucidates the real environmental impact of biorefinery proposals in a specific region. The most relevant conclusion is related to the potential use of CS. The environmental impact was higher in all the proposed biorefinery scenarios compared with the CS mulching at the regional level. This behavior was attributed to the mulching benefits in the soil since CS helps to maintain the soil nutrient balance. A biorefinery/mulching ratio of 70%/30% was elucidated to distribute the CS produced in Sucre, Colombia. This distribution was the best ratio to avoid affecting soil nutrient balance and boost industrial development towards a more sustainable one in Colombia. However, achieving these objectives requires the coordination of CS processors and farmers to manage the distribution and transport of biomass. In the absence of this coordination, correct biomass distribution would be very expensive and would result in lower economic and environmental results. Regarding the biorefinery scenarios, the xylitol and biogas production was the best alternative to be implemented since the cellulose fractionation and upgrading implies higher capital and operating investment costs. Finally, the PB-LCA approach is a methodology that can be extrapolated to any other process and region since the base of the calculations is related to the well-known LCA approach. Therefore, the proposed methodology in this research serves as an insight to quantify the real impact of a conceptually designed process on a specific region using the PB to establish the level of activity transgression.

## Supplementary Information

Below is the link to the electronic supplementary material.

Supplementary file1 (DOCX 157 KB)

## Data Availability

All data generated or analyzed during this study are included in this published article and its supplementary information files.

## Abbreviations

ABE: Acetone butanol ethanol
AOD: Aerosol optical depth
${C}^{inv}$: The fixed capital investment
CapEx: Capital expenditure
CF: Characterization factors
CS: Corn stover
CsF: Chas flow
C5: Xylose
C6: Glucose
DU: Dobson unit
GAMS: General Algebraic Modeling System
GDP: Gross domestic product
GDP_world_: Gross domestic product of the world
GDP_n_: Gross domestic product of the activity *n*
GDP_country_: Gross domestic product of the country
ICV: Impact on control variables
IPCC: The Intergovernmental Panel on Climate Change
ir: The interest rate on the project
Km^3^yr^−^^1^: Cubic kilometer per year
LCA: Life cycle analysis
LCE: Life cycle evaluation
LCI: Life cycle inventory
MIP: Mixed-integer programming
NPV: Net present value
OpEx: Operational expenditures
PB: Planetary boundaries
PB-LCA: Life cycle analysis with planetary boundaries approach
*P*_world_: Population of the world
*P*_country_: Population of the country
*P*_*a*_: Population of the region *a*
Ppm: Parts per million of CO_2_
SOS: Safe operating space
Tg yr^−1^: Teragram per year
TL: Transgression level
TRL: Technological readiness level
VC: Value chain
Wm^−2^: Watts per cubic meter

## References

[CR1] Aghaei S, Karimi Alavijeh M, Shafiei M, Karimi K (2022) A comprehensive review on bioethanol production from corn stover: worldwide potential, environmental importance, and perspectives. Biomass Bioenergy 161:106447. 10.1016/J.BIOMBIOE.2022.106447

[CR2] Ahmed M, Rauf M, Mukhtar Z, Saeed NA (2017) Excessive use of nitrogenous fertilizers: an unawareness causing serious threats to environment and human health. Environ Sci Pollut Res 24:26983–26987. 10.1007/S11356-017-0589-7/TABLES/2 10.1007/s11356-017-0589-7 29139074

[CR3] Alavijeh RS, Shahvandi A, Okoro OV, Denayer JFM, Karimi K (2023) Biorefining of corn stover for efficient production of bioethanol, biodiesel, biomethane, and value-added byproducts. Energy Convers Manag 283:116877. 10.1016/J.ENCONMAN.2023.116877

[CR4] Aristizábal Marulanda V, Cardona Alzate CA, Martín M (2019) An integral methodological approach for biorefineries design: study case of Colombian coffee cut-stems. Comput Chem Eng 126:35–53. 10.1016/j.compchemeng.2019.03.038

[CR5] Awasthi MK, Sarsaiya S, Patel A, Juneja A, Singh RP, Yan B, Awasthi SK, Jain A, Liu T, Duan Y, Pandey A, Zhang Z, Taherzadeh MJ (2020) Refining biomass residues for sustainable energy and bio-products: an assessment of technology, its importance, and strategic applications in circular bio-economy. Renew Sustain Energy Rev 127:109876. 10.1016/J.RSER.2020.109876

[CR6] Balasuriya BTG, Ghose A, Gheewala SH, Prapaspongsa T (2022) Assessment of eutrophication potential from fertiliser application in agricultural systems in Thailand. Sci Total Environ 833. 10.1016/J.SCITOTENV.2022.154993 10.1016/j.scitotenv.2022.154993 35385761

[CR7] Barnosell I, Pozo C (2023) The impacts of the European chemical industry on the planetary boundaries. Sustain Prod Consum. 10.1016/J.SPC.2023.12.006

[CR8] Bbosa D, Mba-Wright M, Brown RC (2018) More than ethanol: a techno-economic analysis of a corn stover-ethanol biorefinery integrated with a hydrothermal liquefaction process to convert lignin into biochemicals. Biofuels Bioprod Biorefin 12:497–509. 10.1002/BBB.1866

[CR9] Berchem T, Roiseux O, Vanderghem C, Boisdenghien A, Foucart G, Richel A (2017) Corn stover as feedstock for the production of ethanol: chemical composition of different anatomical fractions and varieties. Biofuels Bioprod Biorefin 11:430–440. 10.1002/BBB.1755

[CR10] Bessou C, Lehuger S, Gabrielle B, Mary B (2013) Using a crop model to account for the effects of local factors on the LCA of sugar beet ethanol in Picardy region, France. Int J Life Cycle Assess 18:24–36. 10.1007/S11367-012-0457-0/FIGURES/6

[CR11] British Standard (2006) Environmental management-Life cycle assessment-Principles and framework (ISO 14040:2006)

[CR12] Chen X, Li C, Li M, Fang K (2021) Revisiting the application and methodological extensions of the planetary boundaries for sustainability assessment. Sci Total Environ 788:147886. 10.1016/J.SCITOTENV.2021.147886 34134372 10.1016/j.scitotenv.2021.147886

[CR13] Cherubini F (2010) The biorefinery concept: using biomass instead of oil for producing energy and chemicals. Energy Convers Manag 51:1412–1421. 10.1016/J.ENCONMAN.2010.01.015

[CR14] Cole MJ, Bailey RM, New MG (2014) Tracking sustainable development with a national barometer for South Africa using a downscaled “safe and just space” framework. Proc Natl Acad Sci U S A 111:E4399–E4408. 10.1073/PNAS.1400985111/SUPPL_FILE/PNAS.201400985SI.PDF 25294930 10.1073/pnas.1400985111 PMC4210279

[CR15] Culaba AB, Mayol AP, San Juan JLG, Ubando AT, Bandala AA, Concepcion RS, Alipio M, Chen WH, Show PL, Chang JS (2023) Design of biorefineries towards carbon neutrality: a critical review. Bioresour Technol 369:128256. 10.1016/J.BIORTECH.2022.128256 36343780 10.1016/j.biortech.2022.128256

[CR16] Dearing JA, Wang R, Zhang K, Dyke JG, Haberl H, Hossain MS, Langdon PG, Lenton TM, Raworth K, Brown S, Carstensen J, Cole MJ, Cornell SE, Dawson TP, Doncaster CP, Eigenbrod F, Flörke M, Jeffers E, Mackay AW, Nykvist B, Poppy GM (2014) Safe and just operating spaces for regional social-ecological systems. Glob Environ Chang 28:227–238. 10.1016/J.GLOENVCHA.2014.06.012

[CR17] Duan Y, Tarafdar A, Kumar V, Ganeshan P, Rajendran K, Shekhar Giri B, Gómez-García R, Li H, Zhang Z, Sindhu R, Binod P, Pandey A, Taherzadeh MJ, Sarsaiya S, Jain A, Kumar Awasthi M (2022) Sustainable biorefinery approaches towards circular economy for conversion of biowaste to value added materials and future perspectives. Fuel 325:124846. 10.1016/J.FUEL.2022.124846

[CR18] Ethanol producer magazine (2017) An update on 10 cellulosic ethanol projects around the world. Focus on Catalysts 2017:5–6. 10.1016/J.FOCAT.2017.09.031

[CR19] Fan J, Luo R, Liu D, Chen Z, Luo J, Boland N, Tang J, Hao M, McConkey B, Ding W (2018) Stover retention rather than no-till decreases the global warming potential of rainfed continuous maize cropland. Field Crops Res 219:14–23. 10.1016/J.FCR.2018.01.023

[CR20] Fang K, Heijungs R, De Snoo GR (2015) Understanding the complementary linkages between environmental footprints and planetary boundaries in a footprint–boundary environmental sustainability assessment framework. Ecol Econ 114:218–226. 10.1016/J.ECOLECON.2015.04.008

[CR21] Fenalce (2011) Technical aspects of corn production in Colombia (Aspectos Técnicos de la Producción de Maíz en Colombia). https://repository.agrosavia.co/handle/20.500.12324/19418. Accessed 30 Jan 2024

[CR22] Foreign Agricultural Service (2024) Corn | USDA Foreign Agricultural Service. https://fas.usda.gov/data/production/commodity/0440000. Accessed 4 Sep 2024

[CR23] Galán-Martín Á, Tulus V, Díaz I, Pozo C, Pérez-Ramírez J, Guillén-Gosálbez G (2021) Sustainability footprints of a renewable carbon transition for the petrochemical sector within planetary boundaries. One Earth 4:565–583. 10.1016/J.ONEEAR.2021.04.001/ATTACHMENT/D5F8717E-7F87-4C2E-B364-370B57DB7741/MMC2.PDF

[CR24] García Stepien L, Borlandelli MS, Roldán DO, Ibáñez YM (2017) Corn stover: theoretical production of lignocellulosic ethanol in relation to the sowing date (Rastrojo de Maíz: Producción Teórica de Etanol Lignocelulósico en Relación A LA Fecha de Siembra). Buenos Aires

[CR25] Hames B, Ruiz R, Scarlata C, Sluiter A, Sluiter J, Templeton D (2008) Preparation of samples for compositional analysis: Laboratory Analytical Procedure (LAP); Issue Date 08/08/2008

[CR26] Han JS, Rowell JS (1996) Chemical composition of fibers. In: Paper and composites from agro-based resources, pp 83–134

[CR27] Hao DC, Su XY, Xie HT, Bao XL, Zhang XD, Wang LF (2023) Effects of tillage patterns and stover mulching on N2O production, nitrogen cycling genes and microbial dynamics in black soil. J Environ Manage 345:118458. 10.1016/J.JENVMAN.2023.118458 37385196 10.1016/j.jenvman.2023.118458

[CR28] Hauschild MZ, Kara S, Røpke I (2020) Absolute sustainability: challenges to life cycle engineering. CIRP Ann 69:533–553. 10.1016/J.CIRP.2020.05.004

[CR29] Hjalsted AW, Laurent A, Andersen MM, Olsen KH, Ryberg M, Hauschild M (2021) Sharing the safe operating space: exploring ethical allocation principles to operationalize the planetary boundaries and assess absolute sustainability at individual and industrial sector levels. J Ind Ecol 25:6–19. 10.1111/JIEC.13050

[CR30] Hoff H (2021) A safe operating space for New Zealand/Aotearoa: translating the planetary boundaries framework. SEI: stockholm environment institute, Wellington

[CR31] IPCC (2006) 2006 IPCC guidelines for National greenhouse gas inventories. Institute for global environmental strategies (IGES)

[CR32] Jankowski KJ, Neill C, Davidson EA, Macedo MN, Costa C, Galford GL, Maracahipes Santos L, Lefebvre P, Nunes D, Cerri CEP, McHorney R, O’Connell C, Coe MT (2018) Deep soils modify environmental consequences of increased nitrogen fertilizer use in intensifying Amazon agriculture. Sci Rep 8(1):1–11. 10.1038/s41598-018-31175-1 30194382 10.1038/s41598-018-31175-1 PMC6128839

[CR33] Jiang Y, Xie H, Chen Z (2021) Relationship between the amounts of surface corn stover mulch and soil mesofauna assemblage varies with the season in cultivated areas of northeastern China. Soil Tillage Res 213:105091. 10.1016/J.STILL.2021.105091

[CR34] Jiménez MN, Pinto JR, Ripoll MA, Sánchez-Miranda A, Navarro FB (2017) Impact of straw and rock-fragment mulches on soil moisture and early growth of holm oaks in a semiarid area. Catena (Amst) 152:198–206. 10.1016/J.CATENA.2017.01.021

[CR35] Karina Enríquez M, Casas LC, Torres Triana CF, Turriago García JD, Medina MA, Fajardo SM, Monroy DA (2018) Informe del inventario nacional de gases efecto invernadero 1990–2018 y carbono negro 2010–2018 de colombia

[CR36] Kasinath A, Fudala-Ksiazek S, Szopinska M, Bylinski H, Artichowicz W, Remiszewska-Skwarek A, Luczkiewicz A (2021) Biomass in biogas production: pretreatment and codigestion. Renew Sustain Energy Rev 150:111509. 10.1016/J.RSER.2021.111509

[CR37] Khan MFS, Akbar M, Xu Z, Wang H (2021) A review on the role of pretreatment technologies in the hydrolysis of lignocellulosic biomass of corn stover. Biomass Bioenergy 155:106276. 10.1016/J.BIOMBIOE.2021.106276

[CR38] Kim TH, Kim JS, Sunwoo C, Lee YY (2003) Pretreatment of corn stover by aqueous ammonia. Bioresour Technol 90:39–47. 10.1016/S0960-4(03)00097-X 12835055 10.1016/s0960-8524(03)00097-x

[CR39] Leboreiro J, Hilaly AK (2011) Biomass transportation model and optimum plant size for the production of ethanol. Bioresour Technol 102:2712–2723. 10.1016/J.BIORTECH.2010.10.144 21109426 10.1016/j.biortech.2010.10.144

[CR40] Lee EK, Zhang X, Adler PR, Kleppel GS, Romeiko XX (2020) Spatially and temporally explicit life cycle global warming, eutrophication, and acidification impacts from corn production in the US Midwest. J Clean Prod 242:118465. 10.1016/J.JCLEPRO.2019.118465

[CR41] Li P, Cai D, Luo Z, Qin P, Chen C, Wang Y, Zhang C, Wang Z, Tan T (2016) Effect of acid pretreatment on different parts of corn stalk for second generation ethanol production. Bioresour Technol 206:86–92. 10.1016/J.BIORTECH.2016.01.077 26849200 10.1016/j.biortech.2016.01.077

[CR42] Li S, Thompson M, Moussavi S, Dvorak B (2021) Life cycle and economic assessment of corn production practices in the western US Corn Belt. Sustain Prod Consum 27:1762–1774. 10.1016/J.SPC.2021.04.021

[CR43] MinAgricultura (2017) Sucre. Principales Cultivos por Área Sembrada en 2017. https://www.agronet.gov.co/Documents/SUCRE_2017.pdf. Accessed 12 Sep 2023

[CR44] MinAgricultura (2021) Maíz. Dirección de Cadenas Agrícolas y Forestales 2021. https://sioc.minagricultura.gov.co/AlimentosBalanceados/Documentos/2021-03-31%20Cifras%20Sectoriales%20ma%C3%ADz.pdf. Accessed 23 Aug 2023

[CR45] Nilsson M, Persson Å (2012) Can Earth system interactions be governed? Governance functions for linking climate change mitigation with land use, freshwater and biodiversity protection. Ecol Econ 75:61–71. 10.1016/J.ECOLECON.2011.12.015

[CR46] Noppawan P, Lanctôt AG, Magro M, Navarro PG, Supanchaiyamat N, Attard TM, Hunt AJ (2021) High pressure systems as sustainable extraction and pre-treatment technologies for a holistic corn stover biorefinery. BMC Chem 15:1–11. 10.1186/S13065-021-00762-1/TABLES/2 34051832 10.1186/s13065-021-00762-1 PMC8164268

[CR47] Öhgren K, Bura R, Saddler J, Zacchi G (2007) Effect of hemicellulose and lignin removal on enzymatic hydrolysis of steam pretreated corn stover. Bioresour Technol 98:2503–2510. 10.1016/J.BIORTECH.2006.09.003 17113771 10.1016/j.biortech.2006.09.003

[CR48] Ortiz-Sanchez M, Cardona Alzate CA (2022) Analysis of the routes for biomass processing towards sustainable development in the conceptual design step: strategy based on the compendium of bioprocesses portfolio. Bioresour Technol 350:126852. 10.1016/J.BIORTECH.2022.126852 35183725 10.1016/j.biortech.2022.126852

[CR49] Palmeros Parada M, Osseweijer P, Posada Duque JA (2017) Sustainable biorefineries, an analysis of practices for incorporating sustainability in biorefinery design. Ind Crops Prod 106:105–123. 10.1016/J.INDCROP.2016.08.052

[CR50] Pawlak K, Kołodziejczak M (2020) The role of agriculture in ensuring food security in developing countries: considerations in the context of the problem of sustainable food production. Sustainability 12:5488. 10.3390/SU12135488

[CR51] Peñaranda Gonzalez LV, Montenegro Gómez SP, Giraldo Abad PA (2017) Aprovechamiento de residuos agroindustriales en Colombia. Revista de Investigación Agraria y Ambiental 8:141–150. 10.22490/21456453.2040

[CR52] Peters MS, Timmerhaus KD, West RE (2002) Plant design and economics for chemical engineers. 5th edn. McGraw-Hill

[CR53] Prasuhn V (2020) Twenty years of soil erosion on-farm measurement: annual variation, spatial distribution and the impact of conservation programmes for soil loss rates in Switzerland. Earth Surf Process Landf 45:1539–1554. 10.1002/ESP.4829

[CR54] Qin L, Li X, Zhu JQ, Li WC, Xu H, Guan QM, Zhang MT, Li BZ, Yuan YJ (2017) Optimization of ethylenediamine pretreatment and enzymatic hydrolysis to produce fermentable sugars from corn stover. Ind Crops Prod 102:51–57. 10.1016/J.INDCROP.2017.03.026

[CR55] René J, Cardona J (2014) Characterization of colombian creole and indigenous breeds of corn by means of ssr molecular markers (Caracterización de las razas criollas e indígenas de maíz colombiano por medio de marcadores moleculares ssr. Universidad Nacional de Colombia

[CR56] Richardson K, Steffen W, Lucht W, Bendtsen J, Cornell SE, Donges JF, Drüke M, Fetzer I, Bala G, von Bloh W, Feulner G, Fiedler S, Gerten D, Gleeson T, Hofmann M, Huiskamp W, Kummu M, Mohan C, Nogués-Bravo D, Petri S, Porkka M, Rahmstorf S, Schaphoff S, Thonicke K, Tobian A, Virkki V, Wang-Erlandsson L, Weber L, Rockström J (2023) Earth beyond six of nine planetary boundaries. Sci Adv 9. 10.1126/SCIADV.ADH2458/SUPPL_FILE/SCIADV.ADH2458_SM.PDF 10.1126/sciadv.adh2458 PMC10499318 37703365

[CR57] Rockström J, Steffen W, Noone K, Persson Å, Chapin FS, Lambin EF, Lenton TM, Scheffer M, Folke C, Schellnhuber HJ, Nykvist B, De Wit CA, Hughes T, Van Der Leeuw S, Rodhe H, Sörlin S, Snyder PK, Costanza R, Svedin U, Falkenmark M, Karlberg L, Corell RW, Fabry VJ, Hansen J, Walker B, Liverman D, Richardson K, Crutzen P, Foley JA (2009) A safe operating space for humanity. Nature 461(7263):472–475. 10.1038/461472a 19779433 10.1038/461472a

[CR58] Romero-Figueroa JC, Sánchez-Escudero J, Rodríguez-Mendoza MN, Gutiérrez-Castorena MC (2015) Producción de vermicompost a base de rastrojo de maíz (Zea mays l.) y estiércol de bovino lechero. In: agro productividad 8. https://revista-agroproductividad.org/index.php/agroproductividad/article/view/661. Accessed 9 Sep 2024

[CR59] Rueda-Duran CA, Ortiz-Sanchez M, Cardona-Alzate CA (2022) Detailed economic assessment of polylactic acid production by using glucose platform: sugarcane bagasse, coffee cut stems, and plantain peels as possible raw materials. Biomass Convers Biorefin 12:4419–4434. 10.1007/S13399-022-02501-5/FIGURES/7

[CR60] Ryberg MW, Owsianiak M, Clavreul J, Mueller C, Sim S, King H, Hauschild MZ (2018a) How to bring absolute sustainability into decision-making: an industry case study using a Planetary Boundary-based methodology. Sci Total Environ 634:1406–1416. 10.1016/J.SCITOTENV.2018.04.075 29710640 10.1016/j.scitotenv.2018.04.075

[CR61] Ryberg MW, Owsianiak M, Richardson K, Hauschild MZ (2018b) Development of a life-cycle impact assessment methodology linked to the planetary boundaries framework. Ecol Indic 88:250–262. 10.1016/J.ECOLIND.2017.12.065

[CR62] Sala S, Crenna E, Secchi M, Sanyé-Mengual E (2020) Environmental sustainability of European production and consumption assessed against planetary boundaries. J Environ Manage 269:110686. 10.1016/J.JENVMAN.2020.110686 32560978 10.1016/j.jenvman.2020.110686 PMC7315131

[CR63] Serratos Hernández JA (2009) The origin and diversity of corn in the American continent (El origen y la diversidad del maíz en el continente Americano). Ciudad de mexico

[CR64] Shaji A, Shastri Y, Kumar V, Ranade VV, Hindle N (2022) Sugarcane bagasse valorization to xylitol: techno-economic and life cycle assessment. Biofuels, Bioprod Biorefin 16:1214–1226. 10.1002/BBB.2368

[CR65] Sluiter A, Hames B, Ruiz R, Scarlata C, Sluiter J, Templeton D (2008a) Determination of ash in biomass: Laboratory Analytical Procedure (LAP); Issue Date: 7/17/2005

[CR66] Sluiter A, Ruiz R, Scarlata C, Sluiter J, Templeton D (2008b) Determination of extractives in biomass: Laboratory Analytical Procedure (LAP); Issue Date 7/17/2005

[CR67] Sluiter AD, Hayward TK, Jurich CK, Newman MM, Templeton DW, Ruth MF, Evans KW, Hames BR, Thomas SR (2003) Compositional variability among corn stover samples. In: National renewable energy laboratory. https://www.nrel.gov/docs/gen/fy03/33925.pdf. Accessed 27 Jan 2024

[CR68] Steffen W, Richardson K, Rockström J, Cornell SE, Fetzer I, Bennett EM, Biggs R, Carpenter SR, De Vries W, De Wit CA, Folke C, Gerten D, Heinke J, Mace GM, Persson LM, Ramanathan V, Reyers B, Sörlin S (2015) Planetary boundaries: guiding human development on a changing planet. Science 1979:347. 10.1126/SCIENCE.1259855/SUPPL_FILE/STEFFEN-SM.PDF 10.1126/science.1259855 25592418

[CR69] Sun C, Chen L, Zhai L, Liu H, Wang K, Jiao C, Shen Z (2020) National assessment of nitrogen fertilizers fate and related environmental impacts of multiple pathways in China. J Clean Prod 277:123519. 10.1016/J.JCLEPRO.2020.123519

[CR70] Surmeier A, Meyer I, Maleka M (2024) Living wages in global value chains: pitfalls and pathways to successful implementation. Organ Dyn 101109. 10.1016/J.ORGDYN.2024.101109

[CR71] T.A.P.P.I (2006) Acid-insoluble lignin in wood and pulp (Reaffirmation of T 222 om-02). https://www.tappi.org/content/sarg/t222.pdf. Accessed 20 May 2024

[CR72] Tulus V, Pérez-Ramírez J, Guillén-Gosálbez G (2021) Planetary metrics for the absolute environmental sustainability assessment of chemicals. Green Chem 23:9881–9893. 10.1039/D1GC02623B 35002534 10.1039/d1gc02623b PMC8667789

[CR73] United Nations General Assembly (1987) Report of the world commission on environment and development: our common future. https://sustainabledevelopment.un.org/content/documents/5987our-common-future.pdf. Accessed 6 Aug 2024

[CR74] van Kessel C, Venterea R, Six J, Adviento-Borbe MA, Linquist B, van Groenigen KJ (2013) Climate, duration, and N placement determine N2O emissions in reduced tillage systems: a meta-analysis. Glob Chang Biol 19:33–44. 10.1111/J.1365-2486.2012.02779.X 23504719 10.1111/j.1365-2486.2012.02779.x

[CR75] Vargas L, Willemen L, Hein L (2018) Linking planetary boundaries and ecosystem accounting, with an illustration for the Colombian Orinoco river basin. Reg Environ Change 18:1521–1534. 10.1007/S10113-018-1282-1/FIGURES/2 31007598 10.1007/s10113-018-1282-1 PMC6448356

[CR76] Wang H, Wang S, Yu Q, Zhang Y, Wang R, Li J, Wang X (2020) No tillage increases soil organic carbon storage and decreases carbon dioxide emission in the crop residue-returned farming system. J Environ Manage 261:110261. 10.1016/J.JENVMAN.2020.110261 32148320 10.1016/j.jenvman.2020.110261

[CR77] Young MD, Ros GH, de Vries W (2021) Impacts of agronomic measures on crop, soil, and environmental indicators: a review and synthesis of meta-analysis. Agric Ecosyst Environ 319:107551. 10.1016/J.AGEE.2021.107551

[CR78] Zabed HM, Akter S, Yun J, Zhang G, Zhao M, Mofijur M, Awasthi MK, Kalam MA, Ragauskas A, Qi X (2023) Towards the sustainable conversion of corn stover into bioenergy and bioproducts through biochemical route: technical, economic and strategic perspectives. J Clean Prod 400:136699. 10.1016/J.JCLEPRO.2023.136699

[CR79] Zhang D, Shen J, Zhang F, Li Y, Zhang W (2017) Carbon footprint of grain production in China. Sci Rep 7:1–11. 10.1038/s41598-017-04182-x 28663590 10.1038/s41598-017-04182-x PMC5491493

[CR80] Zhang H, Zhang R, Song Y, Miu X, Zhang Q, Qu J, Sun Y (2023) Enhanced enzymatic saccharification and ethanol production of corn stover via pretreatment with urea and steam explosion. Bioresour Technol 376:128856. 10.1016/J.BIORTECH.2023.128856 36907227 10.1016/j.biortech.2023.128856

[CR81] Zhao X, Liang X, Han S, Uryu T, Yoshida T (2014) Successive saccharification and fermentation of cellulosic agricultural residues using a combination of cellulase and recombinant yeast. Sen’i Gakkaishi 70:191–196. 10.2115/FIBER.70.191

[CR82] Zhao Y, Damgaard A, Christensen TH (2018) Bioethanol from corn stover – a review and technical assessment of alternative biotechnologies. Prog Energy Combust Sci 67:275–291. 10.1016/J.PECS.2018.03.004

[CR83] Zhuo S, Peng B, Yan X, Zhang K, Si M, Liu M, Shi Y (2018) Conquering lignin recalcitrance by Pandoraea sp. B-6 to improve co-solvent pretreatment of corn stover. Process Biochem 75:187–193. 10.1016/J.PROCBIO.2018.09.012

