# Supplementary material for: Analysis of planetary boundaries and economic assessment for waste valorization in the context of a biorefinery: case study of the corn value chain in Sucre, Colombia
Source: Environ Sci Pollut Res Int. 2025 Apr 2;33(6):1835–53. doi: 10.1007/s11356-025-36266-x (PMC12960393; doi:10.1007/s11356-025-36266-x)
Supplement: Supplementary file 1 — Supplementary file1 (DOCX 157 KB) [file 11356_2025_36266_MOESM1_ESM.docx]

Table S1. Costs and prices used in economic assessment

| Raw material | | | Ref |
| --- | --- | --- | --- |
| Corn Stover | 0,01 | USD/Ton | this research |
| supplies | | |  |
| water | 0,33 | USD/Ton | (Ortiz-Sanchez et al. 2024a) |
| enzymes | 49,30 | USD/kg |  |
| H2SO4 | 0,17 | USD/Ton |  |
| Lime | 0,13 | USD/Ton |  |
| Ethanol (solvent) | 1,84 | USD/L |  |
| CO2 | 0,48 | USD/kg | (Indiamart 2024a) |
| NaOH(solid) | 251 | USD/Ton | (IndexBox 2024) |
| Activated Carbon | 600 | USD/ton | (Özüdoğru et al. 2019) |
| Products | | |  |
| Biogas | 0,15 | USD/m3 | (Ortiz-Sanchez et al. 2024a) |
| Solid digestate | 7,36 | USD/Ton |  |
| Liquid digestate | 4,16 | USD/Ton |  |
| Butanol | 1,23 | USD/L | (Meramo-Hurtado et al. 2021) |
| Acetone | 1,2 | USD/kg |  |
| Ethanol | 1,3 | USD/L |  |
| Xylitol | 3000 | USD/ton | (Özüdoğru et al. 2019) |
| kraft lignin | 350 | USD/ton | (Poveda-Giraldo et al. 2021) |
| organosolv lignin | 450 | USD/ton |  |
| Biomethane | 0,45 | USD/ton | (Trading Economics 2024b) |
| Utilities | | |  |
| Ethylene glycol | 0,85 | USD/L | (Meramo-Hurtado et al. 2021) |
| low Pressure steam | 7,89 | USD/ton | (Ortiz-Sanchez et al. 2024a) |
| Medium Pressure steam | 8,07 | USD/ton |  |
| Electricity | 0,25 | USD/Kwh |  |
| Cooling Water | 0,042 | USD/m3 |  |
| supervisors | 2,21 | USD/h | this research |
| operators | 1,42 | USD/h |  |

Table S2. Life cycle inventory of the transformers link for corn stover

| **Activity** | **Inlet** | | | **Output** | | |
| --- | --- | --- | --- | --- | --- | --- |
|  | **Item** | **Value** | **Unit** | **Item** | **Value** | **Unit** |
| Pretratament | corn stover | 1.20 | ton/h |  | 796.5 |  |
|  | water | 19.43 | ton/h | solid waste |  | kg/h |
|  | Sulfuric Acid | 394.78 | kg/h |  |  |  |
|  | cooling water | 245.00 | m3/h |  |  |  |
|  | Steam | 2.89 | ton/h |  |  |  |
| Fermentation and separation | lime | 298.04 | kg/h | furfural | 3.3 | kg/h |
|  |  |  |  | water | 17.9 | ton/h |
|  |  |  |  | gypsum | 692.2 | kg/h |
|  | cooling water | 69.57 | m3/h | Xylitol | 173.6 | kg/h |
|  |  |  |  | Sulfuric acid | 0.1 | kg/h |
|  | steam | 8.43 | ton/h | xylose | 4.0 | Kg/h |
|  |  |  |  |  |  |  |
|  | electricity | 37.64 | kwh |  |  |  |

Table S3. Ecoinvent information used

| Products | | |
| --- | --- | --- |
| Maiz_masa | 1 | ton |
| Xylitol_masa | 0.173 | ton |
| Resources | | |
| Water, river, CO | 19.43 | m3 |
| Water, cooling, unspecified natural origin, CO | 245 | m3 |
| Water, river, CO | 0.162 | m3 |
| Water, cooling, unspecified natural origin, CO | 69.57 | m3 |
| Materials/fuels | | |
| Sulfuric acid {RoW}\| production \| Alloc Def, U | 394.78 | kg |
| Glyphosate {RoW}\| production \| Alloc Def, U | 0.155 | kg |
| Pyrethroid-compound {RoW}\| production \| Alloc Def, U | 0.13 | kg |
| Transport, motorcycle, gasoline powered/personkm/RNA | 44.3 | personkm |
| Lubricating oil {RoW}\| production \| Alloc Def, U | 0.318 | kg |
| Gasoline (regular), from crude oil, consumption mix, at refinery, 100 ppm sulphur EU-15 S | 4.55 | kg |
| Nitrogen fertiliser, as N {GLO}\| market for \| Alloc Def, U | 48.7 | kg |
| Diesel {RoW}\| market for \| Alloc Def, U | 13 | kg |
| Phosphorous fertilizer, production mix, at plant/US | 25.97 | kg |
| Potassium fertiliser, as K2O {GLO}\| market for \| Alloc Def, U | 51.95 | kg |
| Sulfur {GLO}\| market for \| Alloc Def, U | 0.029 | kg |
| Sodium {RoW}\| chloride electrolysis \| Alloc Def, U | 0.003 | kg |
| Lime {RoW}\| production, milled, loose \| Alloc Def, U | 298.04 | kg |
| Electricity/heat | | |
| Heat, in chemical industry {RoW}\| steam production in chemical industry \| Alloc Def, U | 6.33 | GJ |
| Heat, in chemical industry {RoW}\| steam production in chemical industry \| Alloc Def, U | 18.49 | GJ |
| Electricity, high voltage {RoW}\| electricity production, hydro, reservoir, tropical region \| Alloc Def, U | 37.64 | kWh |
| Emissions to water | | |
| Furfural | 3.3 | kg |
| Sulfuric acid | 0.1 | kg |
| Final waste flows | | |
| Waste, solid | 796.5 | kg |
| Waste to treatment | | |
| Wastewater, average {RoW}\| market for wastewater, average \| Alloc Def, U | 17.9 | m3 |

**sample for the calculation of impacts following the PB-LCA methodology:**

The base case (B.C) will be taken together with the results of the climate change category presented in **Table 7**. First, it starts by annualizing the functional unit (1kg corn) considered in the LCA analysis according to the limits considered in the analysis. flow of 22,080 tons/year was considered. production can be estimated at a 1:1.2 ratio (corn:CS).

$$Corn_{year}=22,080*\left( \frac{1}{1.2} \right)=18,400 ton/year$$

Using the characterization factors provided by Ryberg et al. (Ryberg et al. 2018a) the impact of the climate change category on the PB control variables is calculated annualizing the result.

| $ICV_{n}=CF_{n}.LCI_{n}$ | Eq. (2) |
| --- | --- |
| $ICV_{climate change}=3.53*{10}^{-13}\left( \frac{w.yr}{m^{2}.kg CO_{2}} \right)*0.49 \left( \frac{kg CO_{2}}{kg corn} \right)*1.84e^{7} \left( \frac{kg corn}{yr} \right)$ |  |
| $ICV_{climate change}=1.8636 e^{-5}\frac{w}{m2}$ |  |

From the equations in Table 6, the SOS was calculated according to the criteria used. We took a world population of 8000 million people, a population in Colombia of 52 million and 1 million in the region of Sucre. Data provided by the International Monetary Fund for world GDP were used (104,476,432 mUSD) and for the GDP of Colombia (363,835 mUSD) ([CSL STYLE ERROR: reference with no printed form.]). The GDP of the Sucre region was taken to represent 0.79% of Colombia's GDP. and that corn farming accounts for 11.4% of the sucre region's GDP. (MinCIT 2024)

$$\boldsymbol{SO}\boldsymbol{S}_{\boldsymbol{n}}\boldsymbol{=}\boldsymbol{SO}\boldsymbol{S}_{\boldsymbol{T}}\mathbf{*}\frac{\boldsymbol{P}_{\boldsymbol{a}}}{\boldsymbol{P}_{\boldsymbol{world}}}$$

$$SOS_{a}=1\frac{w}{m^{2}}*\frac{1}{8,000}=0.000125 \frac{w}{m^{2}}$$

$$\boldsymbol{SO}\boldsymbol{S}_{\boldsymbol{n}}\boldsymbol{=}\boldsymbol{SO}\boldsymbol{S}_{\boldsymbol{T}}\mathbf{*}\frac{\boldsymbol{GD}\boldsymbol{P}_{\boldsymbol{n}}}{\boldsymbol{GD}\boldsymbol{P}_{\boldsymbol{world}}}$$

$$SOS_{b}=1\frac{w}{m^{2}}*\frac{\left( 363,835 mUSD \right)*\left( 0,79\% \right)*\left( 11,4\% \right)}{104,476,432 mUSD}=3.1363e^{-6} \frac{w}{m^{2}}$$

$$\boldsymbol{SO}\boldsymbol{S}_{\boldsymbol{n}}\boldsymbol{=}\boldsymbol{SO}\boldsymbol{S}_{\boldsymbol{T}}\mathbf{*}\frac{\boldsymbol{P}_{\boldsymbol{country}}}{\boldsymbol{P}_{\boldsymbol{world}}}\mathbf{*}\frac{\boldsymbol{GD}\boldsymbol{P}_{\boldsymbol{n}}}{\boldsymbol{GD}\boldsymbol{P}_{\boldsymbol{country}}}$$

$$SOS_{c}=1\frac{w}{m^{2}}*\frac{52}{8,000}*\left( 0,79\% \right)*\left( 11,4\% \right)=5.85e^{-6} \frac{w}{m^{2}}$$

Once the SOS has been calculated, we proceed to calculate the TL and determine the impact of the activity in terms of the PBs.

$$TL_{n}=\frac{ICV_{climate change}}{SOS_{n}}$$

$$TL_{a climate change}=\frac{1.86 e^{-5}\frac{w}{m2}}{0.000125 \frac{w}{m^{2}}}=0,149$$

$$TL_{b climate change}=\frac{1.86 e^{-5}\frac{w}{m2}}{3.13e^{-6} \frac{w}{m^{2}}}=5.943$$

$$TL_{c climate change}=\frac{1.86 e^{-5}\frac{w}{m2}}{5.85e^{-6} \frac{w}{m^{2}}}=3.183$$

Simulation conditions

**Organosolvents:** The modeling of the organosolv pretreatment followed the results obtained in the experimental work of Park et al. (Park et al. 2018) With a solvent mixture 56% self-catalyzed ethanol. Temperature 185 °C. The modeling consisted of the separation stage of the lignocellulosic fractions with the respective fiber washes, followed by the recovery of the solvent (García et al. 2011). Followed a 4:1 w/w ratio of solvents/CS (Nitzsche et al. 2016).

**Enzymatic Hydrolysis:** The simulation followed the methodology proposed by (Ashraf and Schmidt 2018). Where a reaction temperature of 50° C was taken. A ratio of 20 mg of enzyme per gram was used. A solid/liquid ratio of 1:20 was applied (Ortiz-Sanchez et al. 2024b). For the yields for CS with Organsolv pretreatment, were used the experimental results of (Park et al. 2018). The yield of the CS subjected to the dilute acid was taken from the yield of the experimental tests of (Li et al. 2019). For CS that has not been subjected to pretreatment, experimental results of (Wang et al. 2020).

**Alkaline pretreatment:** The main function of alkaline pretreatment is to remove lignin from lignocellulosic material. For the reaction conditions as well as for the pretreatment efficiency, we used as reference the experimental results obtained by (Chen et al. 2009). A 1:8 w/w ratio of alkali/CS was used. The temperature was set at 120 °C for a reaction time of 30 min. For each kg of CS, 6 kg of water is added to the solution.

**Acid pretreatment: Acid** treatment seeks the release of hemicellulose by degrading it into xylose C5 sugars. During this process, secondary reactions take place where other substances are formed, mainly furfural. For the pretreatment modeling, we followed the kinetic model proposed by (Jin et al. 2011). Formulated from experimental tests with CS. A concentration of 2% by weight of sulfuric acid was taken. With a solid/liquid ratio of 1:20. The operating temperature was set at 100 °C.

**ABE: The** results of the fermentation process followed the model presented by (Meramo-Hurtado et al. 2021) and (Darkwah et al. 2018), The operating conditions for the fermentation also followed the results of this author, setting the reactor operating temperature at 37 °C. For the simulation in Aspen Plus of the separation zone of acetone, butanol, ethanol, the model and conditions presented by (Haigh et al. 2018) and (Ezeji et al. 2005).

**Xylitol:** Experimental results are taken from (Mussatto and Roberto 2004) the simulation was guided by the process proposed by (Dasgupta et al. 2021). As well as the crystallization yields for detoxification with CA, we used the results obtained in (Hodge et al. 2009). Reactions involved were taken from (Gerbrandt 2014), the hydrolysate was concentrated to 90 g/l xylose and fermentation was carried out at 30 °C.

**Lignina Kraft:** The LignoBoost process will be used for lignin purification, the amount of CO2 used will be 200kg per ton of lignin (Kienberger et al. 2021) the general process was simulated by following the process diagram presented by (Tomani 2010).

**Anaerobic Digestion:** For the simulation of anaerobic digestion, were followed the model proposed by (Hilby) Following a stoichiometric model, the digestion conditions are taken from (Liu et al. 2018), furthermore, the conditions as the general scheme for methane production by pressure water washing were mainly based on (Barbera et al. 2019).

**Ethanol:** Ethanol fermentation followed a classical scheme with two distillation towers for ethanol concentration until a point close to the azeotropic concentration, then dried to anhydrous point with a molecular sieve stage in conjunction with a fermentation using saccharomyces cerevisiae (Zhao et al. 2018).

Ashraf MT, Schmidt JE (2018) Process simulation and economic assessment of hydrothermal pretreatment and enzymatic hydrolysis of multi-feedstock lignocellulose – Separate vs combined processing. Bioresour Technol 249:835–843. https://doi.org/10.1016/J.BIORTECH.2017.10.088

Barbera E, Menegon S, Banzato D, D’Alpaos C, Bertucco A (2019) From biogas to biomethane: A process simulation-based techno-economic comparison of different upgrading technologies in the Italian context. Renew Energy 135:663–673. https://doi.org/10.1016/J.RENENE.2018.12.052

Chen M, Zhao J, Xia L (2009) Comparison of four different chemical pretreatments of corn stover for enhancing enzymatic digestibility. Biomass Bioenergy 33:1381–1385. https://doi.org/10.1016/J.BIOMBIOE.2009.05.025

Darkwah K, Nokes SE, Seay JR, Knutson BL (2018) Mechanistic simulation of batch acetone-butanol-ethanol (ABE) fermentation with in situ gas stripping using Aspen Plus^TM^. Bioprocess Biosyst Eng 41:1283–1294. https://doi.org/10.1007/S00449-018-1956-6

Dasgupta D, Sidana A, Ghosh P, Sharma T, Singh J, Prabhune A, More S, Bhaskar T, Ghosh D (2021) Energy and life cycle impact assessment for xylitol production from corncob. J Clean Prod 278:123217. https://doi.org/10.1016/J.JCLEPRO.2020.123217

Ezeji TC, Karcher PM, Qureshi N, Blaschek HP (2005) Improving performance of a gas stripping-based recovery system to remove butanol from Clostridium beijerinckii fermentation. Bioprocess Biosyst Eng 27:207–214. https://doi.org/10.1007/S00449-005-0403-7/TABLES/1

García A, Alriols MG, Llano-Ponte R, Labidi J (2011) Energy and economic assessment of soda and organosolv biorefinery processes. Biomass Bioenergy 35:516–525. https://doi.org/10.1016/J.BIOMBIOE.2010.10.002

Gerbrandt KL (2014) The Impacts of Xylitol Production from Hemicellulose Residues: Process Design, Life Cycle, and Techno-Economic Assessment

Haigh KF, Petersen AM, Gottumukkala L, Mandegari M, Naleli K, Görgens JF (2018) Simulation and comparison of processes for biobutanol production from lignocellulose via ABE fermentation. Biofuels, Bioproducts and Biorefining 12:1023–1036. https://doi.org/10.1002/BBB.1917

Hilby E Modeling of Biogas Formation and Utilization using Aspen Plus

Hodge DB, Andersson C, Berglund KA, Rova U (2009) Detoxification requirements for bioconversion of softwood dilute acid hydrolyzates to succinic acid. Enzyme Microb Technol 44:309–316. https://doi.org/10.1016/J.ENZMICTEC.2008.11.007

IndexBox (2024) Caustic Soda Price in Colombia - Charts and Tables - IndexBox. https://www.indexbox.io/search/caustic-soda-price-colombia/. Accessed 3 Aug 2024

Jin Q, Zhang H, Yan L, Qu L, Huang H (2011) Kinetic characterization for hemicellulose hydrolysis of corn stover in a dilute acid cycle spray flow-through reactor at moderate conditions. Biomass Bioenergy 35:4158–4164. https://doi.org/10.1016/J.BIOMBIOE.2011.06.050

Kienberger M, Maitz S, Pichler T, Demmelmayer P (2021) Systematic Review on Isolation Processes for Technical Lignin. Processes 2021, Vol 9, Page 804 9:804. https://doi.org/10.3390/PR9050804

Li J, Zhang H, Lu M, Han L (2019) Comparison and intrinsic correlation analysis based on composition, microstructure and enzymatic hydrolysis of corn stover after different types of pretreatments. Bioresour Technol 293:122016. https://doi.org/10.1016/J.BIORTECH.2019.122016

Liu CM, Wachemo AC, Tong H, Shi SH, Zhang L, Yuan HR, Li XJ (2018) Biogas production and microbial community properties during anaerobic digestion of corn stover at different temperatures. Bioresour Technol 261:93–103. https://doi.org/10.1016/J.BIORTECH.2017.12.076

Meramo-Hurtado SI, González-Delgado Á, Rehmann L, Quinones-Bolanos E, Mehvar M (2021) Comparative analysis of biorefinery designs based on acetone-butanol-ethanol fermentation under exergetic, techno-economic, and sensitivity analyses towards a sustainability perspective. J Clean Prod 298:126761. https://doi.org/10.1016/J.JCLEPRO.2021.126761

MinCIT (2024) Perfiles Económicos Departamentales Departamento de Sucre Perfil económico: Departamento de Sucre Contenido

Mussatto SI, Roberto IC (2004) Kinetic behavior of Candida guilliermondii yeast during xylitol production from highly concentrated hydrolysate. Process Biochemistry 39:1433–1439. https://doi.org/10.1016/S0032-9592(03)00261-9

Nitzsche R, Budzinski M, Gröngröft A (2016) Techno-economic assessment of a wood-based biorefinery concept for the production of polymer-grade ethylene, organosolv lignin and fuel. Bioresour Technol 200:928–939. https://doi.org/10.1016/J.BIORTECH.2015.11.008

Ortiz-Sanchez M, Solarte-Toro JC, Inocencio-García PJ, Cardona Alzate CA (2024a) Sustainability analysis of orange peel biorefineries. Enzyme Microb Technol 172:110327. https://doi.org/10.1016/J.ENZMICTEC.2023.110327

Ortiz-Sanchez M, Solarte-Toro JC, Inocencio-García PJ, Cardona Alzate CA (2024b) Sustainability analysis of orange peel biorefineries. Enzyme Microb Technol 172:110327. https://doi.org/10.1016/J.ENZMICTEC.2023.110327

Özüdoğru HMR, Nieder-Heitmann M, Haigh KF, Görgens JF (2019) Techno-economic analysis of product biorefineries utilizing sugarcane lignocelluloses: Xylitol, citric acid and glutamic acid scenarios annexed to sugar mills with electricity co-production. Ind Crops Prod 133:259–268. https://doi.org/10.1016/J.INDCROP.2019.03.015

Park YC, Kim TH, Kim JS (2018) Flow-Through Pretreatment of Corn Stover by Recycling Organosolv to Reduce Waste Solvent. Energies 2018, Vol 11, Page 879 11:879. https://doi.org/10.3390/EN11040879

Poveda-Giraldo JA, Solarte-Toro JC, Cardona Alzate CA (2021) The potential use of lignin as a platform product in biorefineries: A review. Renewable and Sustainable Energy Reviews 138:110688. https://doi.org/10.1016/J.RSER.2020.110688

Ryberg MW, Owsianiak M, Clavreul J, Mueller C, Sim S, King H, Hauschild MZ (2018) How to bring absolute sustainability into decision-making: An industry case study using a Planetary Boundary-based methodology. Science of The Total Environment 634:1406–1416. https://doi.org/10.1016/J.SCITOTENV.2018.04.075

Tomani P (2010) THE LIGNOBOOST PROCESS. CELLULOSE CHEMISTRY AND TECHNOLOGY Cellulose Chem Technol 44:53–58

Wang Z, He X, Yan L, Wang J, Hu X, Sun Q, Zhang H (2020) Enhancing enzymatic hydrolysis of corn stover by twin-screw extrusion pretreatment. Ind Crops Prod 143:111960. https://doi.org/10.1016/J.INDCROP.2019.111960

Zhao Y, Damgaard A, Christensen TH (2018) Bioethanol from corn stover – a review and technical assessment of alternative biotechnologies. Prog Energy Combust Sci 67:275–291. https://doi.org/10.1016/J.PECS.2018.03.004

Indiamart (2024a) Mild Steel Industrial Carbon Dioxide Cylinder, 150 Bar. https://www.indiamart.com/proddetail/co2-cylinder-27218850333.html. Accessed 3 Aug 2024

Trading Economics (2024b) EU Natural Gas TTF. https://tradingeconomics.com/commodity/eu-natural-gas. Accessed 3 Aug 2024

Report for Selected Countries and Subjects. https://www.imf.org/en/Publications/WEO/weo-database/2023/October/weo-report?c=512,914,612,171,614,311,213,911,314,193,122,912,313,419,513,316,913,124,339,638,514,218,963,616,223,516,918,748,618,624,522,622,156,626,628,228,924,233,632,636,634,238,662,960,423,935,128,611,321,243,248,469,253,642,643,939,734,644,819,172,132,646,648,915,134,652,174,328,258,656,654,336,263,268,532,944,176,534,536,429,433,178,436,136,343,158,439,916,664,826,542,967,443,917,544,941,446,666,668,672,946,137,546,674,676,548,556,678,181,867,682,684,273,868,921,948,943,686,688,518,728,836,558,138,196,278,692,694,962,142,449,564,565,283,853,288,293,566,964,182,359,453,968,922,714,862,135,716,456,722,942,718,724,576,936,961,813,726,199,733,184,524,361,362,364,732,366,144,146,463,528,923,738,578,537,742,866,369,744,186,925,869,746,926,466,112,111,298,927,846,299,582,487,474,754,698,&s=NGDPD,&sy=2021&ey=2028&ssm=0&scsm=1&scc=0&ssd=1&ssc=0&sic=0&sort=country&ds=.&br=1. Accessed 3 Aug 2024c
